# Advances in Nanoarchitectonics: A Review of “Static” and “Dynamic” Particle Assembly Methods

**DOI:** 10.3390/ma17051051

**Published:** 2024-02-24

**Authors:** Karaneh Eftekhari, Bogdan V. Parakhonskiy, Dmitry Grigoriev, Andre G. Skirtach

**Affiliations:** 1 Nanobiotechnology Group, Faculty of Bioscience Engineering, Ghent University, 9000 Ghent, Belgium; karaneh.eftekhari@ugent.be; 2 Multifunctional Colloids and Coatings, Division Life Science and Bioprocesses, Fraunhofer Institute for Applied Polymer Research (IAP), 14476 Potsdam-Golm, Germany; dmitry.grigoriev@iap.fraunhofer.de

**Keywords:** particle assembly, biomaterials, static methods, dynamic methods, self-assembly

## Abstract

Particle assembly is a promising technique to create functional materials and devices from nanoscale building blocks. However, the control of particle arrangement and orientation is challenging and requires careful design of the assembly methods and conditions. In this study, the static and dynamic methods of particle assembly are reviewed, focusing on their applications in biomaterial sciences. Static methods rely on the equilibrium interactions between particles and substrates, such as electrostatic, magnetic, or capillary forces. Dynamic methods can be associated with the application of external stimuli, such as electric fields, magnetic fields, light, or sound, to manipulate the particles in a non-equilibrium state. This study discusses the advantages and limitations of such methods as well as nanoarchitectonic principles that guide the formation of desired structures and functions. It also highlights some examples of biomaterials and devices that have been fabricated by particle assembly, such as biosensors, drug delivery systems, tissue engineering scaffolds, and artificial organs. It concludes by outlining the future challenges and opportunities of particle assembly for biomaterial sciences. This review stands as a crucial guide for scholars and professionals in the field, fostering further investigation and innovation. It also highlights the necessity for continuous research to refine these methodologies and devise more efficient techniques for nanomaterial synthesis. The potential ramifications on healthcare and technology are substantial, with implications for drug delivery systems, diagnostic tools, disease treatments, energy storage, environmental science, and electronics.

## 1. Introduction

Materials science, a diverse realm with applications spanning medicine, engineering, and electronics, embraces a spectrum of practical uses [1]. Nanoarchitectonics plays a pivotal role in shaping and facilitating the arrangement of materials at the nanoscale [2]. Often, such an approach involves the assembly of particles into nanostructured materials, yielding substances endowed with traits distinct from those observed in larger quantities [3]. Nanoarchitectonics empowers scientists to fabricate substances boasting distinct characteristics, a feat achieved through the strategic assembly of particles into nanostructured materials, a strategy that sets these materials apart from their bulk counterparts [4]. The significance of nanoarchitectonics lies in its capability to direct material properties, offering researchers the ability to tailor materials to possess specific attributes, meticulously tailored to suit targeted applications [5]. Particle assembly plays an essential role in the formation of nanostructured materials, representing a crucial process in this domain. By exercising precise control over how particles are arranged, it is possible to produce materials with customized properties tailored to meet specific application requirements. Particle assembly often takes place in the present of organic molecules, thus forming the so-called organic–inorganic hybrid structures [6,7]. In such structures, organic and inorganic constituents or phases complement each other by bringing the missing or lacking functionalities. The importance of particle assembly lies in its ability to yield materials that exhibit outstanding characteristics, which often deviate from the typical traits found in bulk materials [8]. Collectively, particle assembly serves as a potent instrument, driving the production of advanced materials characterized by unparalleled properties. These materials hold significant promise for enhancing human health and overall well being, as demonstrated by their diverse applications across various scientific disciplines [9].

Particle assembly techniques can be considered as those based on thermodynamics, “static” and “dynamic”, where the former can denote a spontaneous process in which particles arrange themselves into ordered structures or patterns, eliminating the need for external energy, and “dynamic” methods can be defined as those methods using external energy or stimuli to control particle assembly. One notable illustration of static is self-assembly, wherein particles autonomously arrange themselves into ordered structures determined by factors like their shape, size, or chemical characteristics. This phenomenon leads to spontaneously organizing smaller components into more extensive, meticulously organized patterns [10,11]. In contrast, dynamic methods rely on energy or stimuli to guide the assembly process [12,13]. A technique of note is a directed assembly, which relies on the utilization of external forces, such as electric or magnetic fields, to guide particle assembly toward predefined structures [7]. It is noteworthy that both static and dynamic methods exhibit distinct advantages and limitations. Static approaches offer the advantage of relative simplicity and ease of implementation, often capitalizing on inherent particle properties to drive the process. However, a limitation of static methods lies in their potential constraints on structural diversity, as the final configuration is contingent on the properties of the particles involved [9]. Dynamic particle assembly methods offer advantages like versatility [14] with various materials, mass production scalability [15], and precise assembly control [16]. They leverage self-organization principles, simplifying manufacturing and enabling the creation of responsive materials. However, achieving stability in assembled structures is challenging. Some methods may have high energy consumption, emphasizing the need for ongoing efforts to improve efficiency and sustainability in these technologies [17]. The choice between static and dynamic methodologies depends on the application and the desired properties of the resulting material.

This manuscript explores the details of assembly techniques, particularly focusing on their applications in biomaterial sciences. It systematically addresses the challenges associated with controlling material arrangement and orientation, examining both static and dynamic methods. The challenges of static methods lie in potential constraints on structural diversity, while dynamic methods may face issues related to stability and energy consumption. The contributions of this study lie in providing a comprehensive overview of various assembly techniques, from self-assembly to directed assembly and shear-driven assembly, showcasing their advantages, limitations, and nanoarchitectonics principles. Overall, this manuscript contributes significantly to advancing the understanding of particle assembly for biomaterial sciences, offering valuable insights into future challenges and opportunities.

## 2. Self-Assembly

The self-assembly approach entails the organization of pre-synthesized nanoparticle building blocks into desired architectural structures using a variety of weak interactions [18]. A profound comprehension of molecular assembly methods has unveiled conventional molecules’ limitations in performing specific, advanced, or intricate functions. In these cases, the need arises for an organized multimolecular system to function cooperatively [10]. This drives the need for exploration of the molecular-level interactions between molecules, giving rise to the emergence of supramolecular chemistry. Within this field, the focus centers on molecular interactions, encompassing a range of forces such as ion–ion interactions, ion–dipole interactions, dipole–dipole interactions, van der Waals forces, hydrophobic interactions, electrostatic attractions, hydrogen bonding, π-π stacking of aromatic rings, cation–π interactions, and anion–π interactions. The “π” in π-π stacking refers to the interaction between the pi orbitals of aromatic rings, which is a key factor in stabilizing structures in molecular biology and chemistry. π-π stacking occurs when these orbitals overlap, typically found between aromatic rings, leading to attractive forces that influence molecular conformation. Cation–π interactions involve the electrostatic interaction between a cation and the electron cloud of an aromatic system, while anion–π interactions are similar but involve an anion. These interactions are crucial in various biological processes and synthetic applications [19,20,21].

The concept of supramolecular assembly, a term introduced by Lehn after his pioneering work on host–guest self-assembly in 1987 [22], refers to the spontaneous creation of distinct nanostructures [23]. These structures come into being through dynamic covalent interactions and various non-covalent intermolecular forces [24]. This process takes inspiration from biological systems and has found extensive applications, particularly in biomedicine [25].

Examples of supramolecular assemblies are abundant in nature. For instance, the supramolecular arrangement of phospholipids in cellular membranes and actin in eukaryotic cytoplasm results from this process [26]. In biological systems, supramolecular assembly assumes a critical role in numerous functions, including delineating environmental boundaries, facilitating molecular transport and release, and mediating cell–extracellular interactions and communication.

An essential technique within supramolecular chemistry is self-assembly, a process through which molecules or other assembly elements spontaneously arrange themselves into ordered structures via weak interactions based on specific molecular recognition modes [19,20]. Introducing and utilizing self-assembly has markedly improved the efficiency of constructing intricate molecules. It is now possible to synthesize relatively straightforward monomers through self-assembly, where the binding sites are inherently embedded within the monomer’s structure. These monomers depend on the recognition of bonding sites to facilitate a “procedural” self-assembly process [27], spontaneously forming complex, highly selective, reversible molecules.

This process is governed by molecular interactions that seek thermodynamic equilibrium and a decrease in the system’s free energy [15]. It entails the conversion of disordered molecular entities into organized structures through specific local interactions among the components. A compelling instance of supramolecular self-assembly is DNA origami [9]. This technique involves the nanoscale folding of DNA to fabricate arbitrary two- and three-dimensional shapes. The interactions between complementary base pairs, dictated by their specific sequences, make DNA an effective construction material [28]. The procedure entails folding a long single strand of viral DNA, typically the 7249 bp genomic DNA of the M13 bacteriophage (a specific filamentous bacteriophage), facilitated by numerous smaller “staple” strands. These shorter strands bind to the longer strand at various locations, forming a predetermined two- or three-dimensional shape. Examples range from a smiley face and a coarse map of China to various three-dimensional structures, like cubes [29].

Chemical self-assembly is a process similar to that in biology, wherein molecules are brought together to form structures, and it is extensively studied, especially in supramolecular chemistry. Self-assembly is not just limited to traditional chemistry; it also involves how biomolecules and inorganic substances assemble to create structures [15].

Self-assembly often relies on equilibria and free-energy changes. However, making complex, asymmetrical, or hierarchical structures through basic self-assembly is usually challenging. In contrast, biological systems often use energy-driven processes far from equilibrium. These processes are effective at creating complex, uneven, or hierarchical structures. So, incorporating non-equilibrium and irreversible aspects into traditional self-assembly could help create highly advanced functional materials [12]. The formation of lipid bilayers is a classic illustration of molecular self-assembly. Lipid bilayers are primarily composed of phospholipids, i.e., amphiphilic molecules. These molecules have a hydrophilic phosphate head and two hydrophobic hydrocarbon tails. When these lipids self-assemble, they form bilayer structures, forming phospholipid membranes [30,31]. Biological membranes, in contrast, are not exclusively made up of phospholipids. They also consist of a mixture of other lipids, such as glycolipids and cholesterol. The self-assembly process is driven by the lipids’ amphiphilic nature. The hydrophilic heads are attracted to water, while the hydrophobic tails are repelled by it. In an aqueous environment, these molecules spontaneously arrange themselves into a bilayer structure to minimize the exposure of the hydrophobic tails to water. This results in forming a stable and flexible barrier, serving as the fundamental structure for cellular membranes [32]. Structural formations, including self-assembly, are a common occurrence in biological systems. These principles can be applied when designing various molecular assemblies, especially those with dynamic functions and potential applications in the field of biomedicine. For example, manipulating hydrogen bonding behaviors in short oligopeptides makes it possible to achieve a wide range of structural variations and dynamic properties in their assemblies. The following section provides examples of various systems involving oligopeptides’ self-assembly and their derivatives. These examples highlight significant contributions from biology and biomedicine [9,15].

Nanocrystal self-assembly is another fascinating field [33]. It involves coaxing colloidal nanocrystals to form ordered superlattices, often triggered by processes like solvent evaporation [33]. Practically, this approach has vast potential for material design [33]. It allows the creation of intricate three-dimensional patterns of functional materials with sub-nanometer precision using relatively simple equipment [33].

A nanocrystal superlattice is an array of inorganic objects separated by layers of surface ligands. Expanding the set of ligands with the goal of targeting desired superlattice structures and properties enables novel biomedical and optoelectronic applications [34]. Emerging ligand chemistry and compact inorganic ligands offer unique properties and programmable superlattices [35,36]. Traditional ligands, like oleic acid, can also enhance nanocrystal properties [37]. Multiple self-assembly techniques were employed to prepare nanocrystal superlattices [38]. Among them, the main methods are evaporation-based nanocrystal self-assembly and destabilization-based nanocrystal self-assembly [39,40].

Evaporation-based nanocrystal self-assembly involves solvent evaporation to create two-dimensional nanocrystal superlattice thin films [41]. This method is commonly employed in the late stages of solvent drying when nanocrystals become more densely packed due to decreasing solvent volume [39]. Excess surfactant can be added to enhance the formation of long-range-ordered superlattices [39]. Various strategies include drop-casting, deposition on a surface, vial-based assembly, doctor blade casting, and the use of polar liquids [42,43,44,45,46]. These techniques offer flexibility in controlling superlattice formation and film thickness [38].

On the other hand, destabilization-based nanocrystal self-assembly results in three-dimensional superlattice structures, such as platelets, polyhedra, or spheres [47]. It relies on attractive interactions between nanocrystals induced by the controlled diffusion of the nonsolvent, heating to enrich nonsolvent components, or gravitational sedimentation [40,47,48]. The shape of the resulting superlattice polyhedron is determined by thermodynamic principles and surface energies. Functionalizing nanocrystal surfaces with light-triggered molecular switches or disrupting surfactant bilayers can also lead to specific superlattice morphologies [49,50].

Both evaporation-based and destabilization-based approaches offer distinct pathways to control the dimensions and shapes of these nanocrystal superlattices, contributing to the development of advanced materials and technologies.

## 3. Static Assembly Methods

As mentioned before, static assembly methods denote a spontaneous process in which particles arrange themselves into ordered structures or patterns, eliminating the need for external energy. This phenomenon results from particle interactions thriving to reach a state of thermodynamic equilibrium and minimize the system’s free energy [8]. Static self-assembly leverages nanoparticle interactions to attain a free-energy minimum. In solutions, this is a product of molecular random motion and the affinity of their binding sites for each other. This process can transpire across all size scales and is considerably slower than dynamic self-assembly due to its dependence on the random chemical interactions between particles. In this context, the term “static” signifies that these methods do not necessitate an external energy source. Instead, they depend on inherent properties, like gravitational potential energy or electrostatic forces [26]. This section explores various static assembly methods, including layer-by-layer assembly, template-assisted assembly, and polymer brushes.

### 3.1. Layer-by-Layer Assembly

Layer-by-layer (LbL) assembly is a widely employed technique for coating different types of substrates with various materials, like colloidal particles, polymers, biomolecules, and even cells. This method offers exceptional control and versatility compared to other methods of depositing thin films, making it valuable in research and industrial applications [51,52].

In material science, the process of depositing materials by layers, particularly polyelectrolyte multilayers, has gained significant attention. This technique involves carefully placing materials on a surface and has applications in various fields, including nanotechnology, biotechnology, and environmental science [53]. Initially, the layer-by-layer method was applied for the assembly of particles by Iler [54], but it is the assembly of polymers by the layer-by-layer approach has a larger resonance in the scientific community [55,56,57].

Polyelectrolytes are charged polymers containing electrolyte groups in their repeating units. The charged nature of these polymers allows them to interact with one another and other substances, leading to the formation of multilayered structures [58]. The construction of polyelectrolyte multilayers is accomplished through the LbL assembly. This technique involves the alternating deposition of positively and negatively charged polyelectrolytes onto a substrate, resulting in a multilayered structure where each layer consists of either poly-cations or poly-anions [59]. Polyelectrolytes can undergo self-organization into complex structures when combined with oppositely charged polyelectrolytes [60]. This property proves beneficial in the development of layer-by-layer materials. The ability to manipulate thickness is important in optimizing various practical aspects of LbL materials, including enhancing optical device transparency, encapsulating drug retention, altering surface wetting properties, and improving adhesion [61]. It is important to note that, despite three decades of research into the assembly of polyelectrolyte multilayers through the LbL method, there remains an ongoing debate regarding the physical mechanisms governing various dependencies of adsorbed quantities on the number of bilayers [62]. This discussion critically examines the fundamental aspects of polyelectrolyte multilayer growth. Notably, the primary distinctions in growth mechanisms are typically linked to the diffusion of adsorbing molecules within the multilayer structure [62].

Creating multilayers through LbL deposition is a versatile technique, offering efficient control over deposition parameters. By modifying deposition conditions, such as pH, temperature, and ionic strength, or by varying the attributes of the polyelectrolytes employed, including molar mass, charge density, linear or branched structure, and the solvent used, it becomes possible to generate materials with diverse structures and properties. In the early 2000s, numerous studies delved into the impact of pH, temperature, ionic strength, and other variables on the LbL assembly of polyelectrolytes, illustrating the capability to tailor the characteristics of polyelectrolyte multilayers [15].

Normally, there is no diffusion in assembled polyelectrolytes [63]. However, some efforts by Elbert and colleagues aimed at offering a consistent physical explanation for the distinctions observed in multilayer growth were undertaken [64]. Their research revealed that polyelectrolytes can diffuse within the multilayer structure, but this was observed in weakly interacting polyelectrolytes. However, the diffusion process exhibits slight variations depending on the type of multilayer growth [64].

In the case of linear growth multilayers, the adsorption process initiates with the deposition of molecular polyelectrolyte layers. Subsequently, some chains diffuse into the inner region of the multilayer. This phenomenon results in a significant intermingling of the layers, creating polyelectrolyte blend layers. This observation aligns with the theoretical framework proposed by Subbotin and Semenov, utilizing a mean-field approach [26]. The origin of non-linear growth proposed by Elbert et al. aligns with the works by Picart et al. [65]. They associated the emergence of non-linear growth with an in-and-out diffusion of at least one of the polyelectrolytes within the multilayer structure, neglecting any role of diffusion in linear growth films. This can be attributed to the absence of control experiments involving linear growth multilayers. Guzmán et al. [66], through a combination of structural characterization of the multilayers and a rigorous analysis of the adsorption kinetics of the polyelectrolyte layers, demonstrated that interdiffusion is not the characteristic difference between linear and non-linear growth. They suggested that an increase in film roughness upon layer deposition may be a crucial driving force for non-linear growth emergence, which may be correlated with Elbert et al.’s proposed deposition of coacervates. Therefore, an increase in roughness may be associated with an increase in available adsorption area, leading to an increase in polyelectrolyte deposited per bilayer, which allows for faster multilayer growth than linear. Many applications have been already shown of this technique [67,68], and its extension is foreseen in the future.

The knowledge accumulated in the area of assembly of polymers has been recently applied to layer-by-layer assembly of particles [69,70], where interesting applications are proposed. Such assembly has allowed to produce a capsule-based nanoparticles that can be used for remotely induced cell killing and release [71]. In addition, label-free sensors based on surface-enhanced Raman scattering have been shown to achieve magnifications of up to 10^6^ times.

### 3.2. Template-Assisted Assembly

One crucial technique in achieving the precise assembly of nanoparticles and nanostructures is template-assisted assembly, where specialized substrates known as templates serve as architectural scaffolds for organizing nanoscale components. Initial template treatment could determined preprocess requirements. 

**Figure 1:**
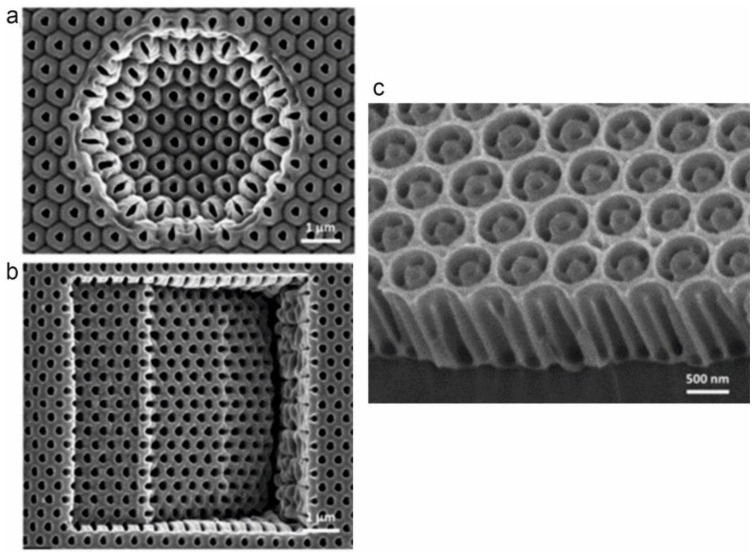
(**a**,**b**) SEM images of anodized nanopore arrays across (**a**) a hemispheric-shaped aluminum surface and (**b**) a staircase-shaped surface. The interpore distance between the nanopores is 350 nm and 200 nm, respectively [76]. (**c**) SEM image of a hierarchical AAO structure by the NSL technique. The period of the pre-patterned aluminum substrate is 680 nm. Reprinted with permission from Ref. [77].

For preprocessed templates, researchers have employed various terminologies, such as porous alumina templates, porous anodic aluminum oxide (AAO) (Figure 1c), nanoporous alumina, nanohole alumina arrays (NAAs), or nanoporous anodized alumina platforms (Figure 1a,b), to describe these templates when harnessing them for the fabrication of nanoparticle arrays [16]. These substrates form through an electrochemical process known as anodic oxidation. A noteworthy characteristic of these porous alumina templates is their remarkable ability to exhibit a high degree of structural regularity, organized into arrays of nanoholes [72].

These tiny apertures span a range of dimensions, ranging from tens to hundreds of nanometers. It is crucial to recognize that these nanohedral structures possess essential properties to serve as matrices or templates, which is essential for creating various nanoarchitectures [73]. The intricate strategy is instrumental in the fabrication of nanoparticle arrays using the templates mentioned above. It is centered on the judicious substance infusion into the nanoscale apertures intrinsic to the porous alumina template. This strategic infusion process precipitates the emergence of nanoarchitectures of varying types, among which are the notable instances of nanowires and nanotubes. The dexterous choice of substances earmarked for this infusion is of paramount significance, as this discerning selection fundamentally arbitrates the attributes encapsulated within these resultant nanostructures [2,10].

Nanoparticle arrays hold promising applications across diverse fields. They can serve as sophisticated photonic structures, finding utility in structural coloration and advanced optical biosensing [70,74,75]. Furthermore, they offer the potential to develop electronic nano-devices and chemical/biological sensors. Porous alumina templates are a versatile and potent resource for creating these nanoparticle arrays, thus unlocking fresh possibilities in nanotechnology [29].

The process of aluminum anodization, initially explored for its surface protection properties, has more recently gained substantial recognition for its capacity to create nano-devices and finely tailored, well-ordered nanostructures. The shift in research emphasis from protective applications to the design of nanostructures highlights its evolving importance. The selected substrate, aluminum, undergoes electrochemical oxidation to produce AAO [78]. This intricate transformation occurs within a specialized electrochemical cell following a dual-electrode arrangement, with aluminum as the working electrode. The critical determinant of the outcome in this transformation lies in the composition of the electrolyte, providing the possibility for forming two distinct categories of anodic oxides. These include the compact, nonporous anodic aluminum oxide and the intricate, porous alumina films [12]. The emergence of nanoporous AAO membranes transpires under the aegis of a more acidic milieu, strategically calibrated to elevate the anodic oxide barrier layer’s solubility marginally. A significant development manifests within this context: the familiar nonporous barrier layer is concomitantly established upon applying a specific potential. Yet, the synergy between heightened solubility within this layer and the acidic environment transmutes the growth trajectory, ushering in the advent of the porous anodic oxide layer. The orchestrated interplay between environmental pH and barrier layer solubility ultimately governs the intricate transformation into the nanoporous domain [79].

In 2016, Jin and colleagues [62] made significant progress by creating three complex branched structures. They combined the steady-state growth of branched pores with pre-patterned curved aluminum substrates. This amalgamation technique, previously described by Zakeri and team [80], results in tree-like porous formations along the boundaries of the patterned cells. These structures are categorized into three distinct types. (1) Type 1 involves the formation of a 3D interconnected branched assembly. (2) Type 2 leads to a self-sustaining architecture of AAO nanowires. (3) Type 3 materializes into what the researchers have labeled a supportive AAO skeleton framework. A rhythmic alternation of anodizing potential is employed to achieve Type 1, the 3D interconnected branched pores oscillating between 50 and 30 V in a medium of 0.3 M oxalic acid. A 35 min etching process follows this. For the subsequent fabrication of Types 2 and 3, a fixed voltage (30 and 40 V) is applied, with varying etching durations. Types 2 and 3 form AAO nanowires, achieved through etching periods ranging from 31 to 33 min and 43 min, respectively. These intricately branched configurations come into existence through this orchestrated interplay of voltage, etching duration, and the unique characteristics of the substrates.

As mentioned before, the construction of alumina membranes offers a versatile array of porous configurations; at the forefront of these morphological innovations reside four principal archetypes: the linear nanowires characterized by their direct trajectory, the longitudinal nanowires, which flaunt varying pore diameters along their extent, the intricately branched nanowires that traverse diverse spatial domains, and the 3D nano-networks constructed through the interconnection of individual nanowires. A rich tapestry of structural diversity unfolds within this orchestrated array of design choices, inviting the assembly of novel constructs with unique properties and functionalities [10,62,81].

In the context of emulsion formation, the use of a preprocess template is not incorporated in Pickering emulsions. In this case, particle assembly methods for emulsion formation have advanced significantly, allowing for the synthesis of small monodisperse particles with various shapes and surface coatings. These particles can be either surface active or amphiphilic, with specific areas designed for oil or water affinity, depending on the coating (e.g., alkylsilane or fluorocarbon). The stability of simple emulsions created with these particles is highly dependent on wettability at the oil–water interface. One of the methods for preparing monodisperse solid-stabilized emulsions involves sequentially replacing surfactant with particles at drop interfaces using dialysis. Additionally, upon spreading, the particles form a 2D film with a tendency toward hexagonal packing, and over time, they diffuse, form clusters, and eventually create a large particle network. This process exhibits a transition from diffusion-limited to convection-limited cluster aggregation, influenced by the attractive energy at the particle contact points [82].

### 3.3. Polymer Brushes for Surface Assembly

Polymer brushes, derived through the terminal attachment of chains to flat or curved surfaces, exhibit either organic or inorganic characteristics. Research has demonstrated that nanoparticles possessing a strong affinity for polymers engage with polymer brushes without undergoing aggregation. However, in cases where the interaction between polymer brushes and nanoparticles is weak, aggregation may occur [83]. Achieving adjustability in the properties of hybrid organic–inorganic brushes is a widely sought-after and frequently applied objective. The functionalization of polymeric brushes by nanoparticles occurs at the interface, where nanoparticles can either adsorb onto the surface of the brushes or be incorporated into the brushes. Factors such as pH, temperature, solvent, and ionic strength play a role in influencing this process [84,85]. The reversible process of swelling and shrinking in polymer brushes allows for the exposure or concealment of nanoparticles within the interior of the brushes. Various nanoparticles, including metal Pt, Ag, Au, and semiconductor CdSe, have been incorporated into brushes, resulting in the formation of hybrid organic–inorganic structures and predominantly serving sensor-like functions [86].

**Figure 2:**
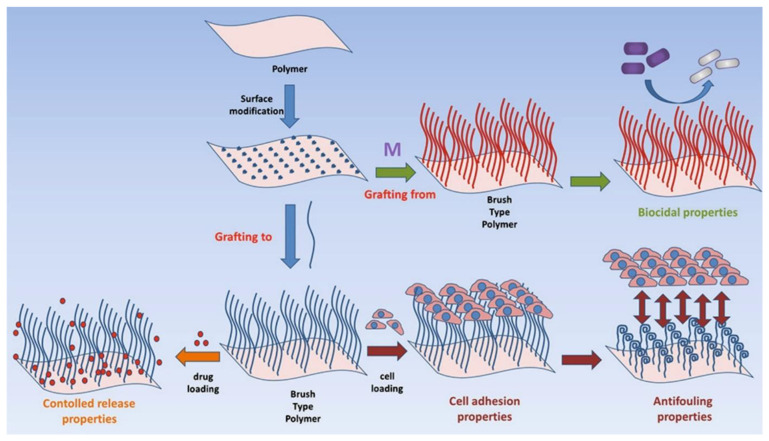
From initial surface modification to advanced biomedical applications: an illustrative journey showcasing the transformative potential of polymer grafting techniques, highlighting controlled release, cell adhesion, and antifouling properties for improved healthcare solutions [87].

Advancements from fundamental surface modifications to intricate biomedical applications using polymer grafting techniques present groundbreaking opportunities in both material science and the healthcare sectors. The versatile and manageable traits of polymer brushes open a diverse range of applications, including controlled drug release, enhanced cell adhesion, and antifouling properties (Figure 2). This catalyzes innovation in the field of medicine.

## 4. Dynamic Assembly Methods

Dynamic techniques involve using energy or external stimuli to control particle assembly. They offer precise control over material structure through external forces [12], as seen in directed assembly, where electric or magnetic fields guide particles into specific arrangements—a common dynamic method. However, dynamic methods can be complex, requiring advanced equipment and techniques [88]. In this section, we will explore various dynamic assembly methods, including directed assembly, shear-driven assembly, and spin coating.

As mentioned before, dynamic methods for particle assembly hold significant promise in the creation of unique and functional materials with customized properties. Nevertheless, these techniques also present notable challenges, including the need to control the fundamental physical processes, fine-tune process parameters, and upscale production [89].

The interface between a material and the surface is important for numerous applications, including microelectronics, displays, sensing, microarrays, photovoltaics, and catalysis. This significance arises from the crucial role that surface functionalization, modification, and patterning play in determining the performance of materials in these diverse applications [90].

Microcontact printing is an illustrative example of a dynamic method employed in this context. This technique utilizes an elastomeric stamp to imprint patterns of self-assembled monolayers (SAMs) of ink onto the surface of a substrate through conformal contact. Creating the stamp involves replicating a master template produced through microlithography. The next step in the process involves coating the stamp with a solution containing ink molecules, often thiols, capable of forming SAMs on the substrate [91]. The stamp is then pressed against the substrate, facilitating the transfer of ink molecules to the areas where the stamp and substrate come into direct contact. An advantage of this method is that the stamp can be reused multiple times. This is achieved by simply replenishing the ink reservoir within the bulk of the stamp [92].

Microcontact printing, a versatile technique, can pattern a wide range of materials, including polymers, proteins, nanoparticles, and DNA. Likewise, it accommodates diverse substrates, including gold, silicon, and glass. Beyond patterning, this method is valuable for creating patterns of lipid bilayers or cell adhesion molecules on surfaces, particularly in cell biology studies. There are numerous advantages of microcontact printing. It offers cost effectiveness, high resolution, and versatility across various applications and materials. However, like any technique, it comes with certain limitations. These include the potential for stamp deformation during the process, the possibility of ink molecule diffusion affecting pattern precision, and the risk of substrate contamination impacting overall result quality [14].

Directed assembly techniques are employed in the mass production of micro- and nano-devices and materials. These methods provide precise control over the assembly of micro- and nanoparticles, enabling the creation of complex and highly functional devices or materials. One specific form of directed assembly is directed self-assembly (DSA), which utilizes the morphology of block copolymers to generate lines, spaces, and hole patterns, allowing for precise control over feature shapes. This process relies on surface interactions and polymer thermodynamics to determine the final pattern shapes [93]. In 2017, a collaborative team from the Massachusetts Institute of Technology, the University of Chicago, and Argonne National Laboratory developed a method to control surface interactions for achieving sub-10 nm resolution. This breakthrough was accomplished using a vapor-phase deposited polymeric top layer on the block copolymer film [94].

DSA is not a standalone process; it is integrated with traditional manufacturing methods to achieve cost-effective mass production of micro- and nanostructures. The primary industries that use directed self-assembly are the semiconductor and hard drive industries. In the semiconductor industry, DSA enhances resolution, allowing for incorporating more gates and improving overall performance. On the other hand, the hard drive industry utilizes DSA to manufacture “bit patterned media” tailored to specific storage densities [95].

Directed assembly finds a wide range of applications at the microscale, with uses spanning from tissue engineering to polymer thin films. In tissue engineering, the directed assembly has supplanted the scaffolding approach by controlling the positioning and organization of different cells, effectively arranging the “building blocks” of tissue into desired microstructures [96].

With the development of nanotechnology, directed assembly offers methods for organizing various materials, including molecules, polymers, and building blocks, to form precise nanostructures with diverse applications. For example, directed assembly plays a crucial role in the peptide self-assembly process and application into nanotubes, with single-wall carbon nanotubes serving as a prominent example. These nanotubes are a graphene sheet seamlessly wrapped into a cylindrical structure [96].

### 4.1. Shear-Driven Assembly

Shear-driven assembly (SDA) is an intriguing field within microfluidics and nanofluidics, offering a unique approach to particle assembly in fluidic environments. At its core, SDA relies on harnessing the potent shear forces that arise when two flat surfaces undergo relative translation, impelling both the fluid and the suspended particles, such as micro- or nanoparticles [97]. Within microfluidic devices, SDA is essential in generating fluid flows within micro- and nanochannels. Unlike conventional methods, like pressure-driven and electro-osmotic flows, which may experience limitations, like pressure drops and Joule heating issues, SDA shines with its ability to produce fluid flows characterized by a linear profile. This feature remains consistent even when channel dimensions vary [98].

By capitalizing on shear forces, SDA provides unparalleled control over particle manipulation, allowing for meticulous control over their orchestrated assembly. This capability carries profound implications across a spectrum of domains, particularly in creating ultrathin, sub-5 nm structures that can stand independently. This remarkable feat is achieved through a self-assembly process driven by solvent extraction within the confines of a microfluidic channel [99].

The landscape of shear-driven assembly is ever-evolving, with researchers tirelessly refining flow-driving methodologies and exploring uncharted application areas. Continuous exploration marks this scientific landscape, with aspirations to optimize methods and unearth novel domains of utility shining brightly. As comprehension of shear phenomena operating on micro- and nanoscales undergoes perpetual expansion, so does the panorama of potential applications that this enthralling technology promises to unveil [100].

Microfluidic devices often employ techniques to facilitate fluid flow, categorized as active or passive. Active microfluidics involves manipulating and transporting biological samples, along with their subsequent analysis, utilizing external power sources or fields, various actuators, kinetics, and static mechanisms. These include peristaltic pumps, electro-wetting, electro-osmotic pumps, and centrifugal and magnetic pumps [101]. However, using such active microfluidics introduces complexities in terms of device structure and size, necessitating augmented human resources and thereby diminishing the feasibility of integrating active microfluidics with lab-on-a-chip (LOC) and point-of-care applications [102].

In response to these limitations, passive microfluidics has gained prominence, offering an alternative approach to sample manipulation. Relying on fluid properties and passive mechanisms, passive microfluidics obviates the need for external power sources. It effectively leverages common laboratory instruments, like micropipettes, and medical devices, like syringe pumps, emerging as a favored choice in contemporary research initiatives due to its simplicity, ease of manufacturing, and independence from actuators or external power supplies [103,104]. Various techniques have been employed within the domain of microfluidics and LOC devices to realize passive operations. These techniques encompass surface tension, pressure-driven mechanisms, osmosis, capillary action, gravity-induced flow, vacuum suction, and hydrostatic pressure. Each of these approaches possesses distinct advantages and disadvantages, setting the stage for a rich exploration into the realms of fluid dynamics and particle manipulation [105].

A common characteristic among self-assemblies driven by chemical fuel sources is their unique kinetic behavior: rapid activation followed by delayed deactivation. These systems have catalyzed the exploration of diverse avenues, including the emergence of novel domains, like feedback-controlled non-Newtonian fluids, and the development of pulsating macromolecular materials and chemical-fueled dynamic covalent bond systems [106,107]. For instance, an illustrative case involves the fusion of a shear-driven fluid with a biocatalytic urea urease switch, which is poised to ignite a new trajectory within non-equilibrium chemistry. In this context, a gamut of energy forms harmonizes, yielding materials with lifelike attributes. Dissipative assembly, a relatively rare phenomenon in macromolecular science, holds particular allure, promising to unveil chemical evolution dynamics under experimental scrutiny [108].

#### 4.1.1. Fluidic Assembly

The field of research dedicated to the manipulation of fluid flow for the organization of particles has gained significant traction, primarily owing to its diverse applications. One innovative approach involves using ultraviolet light to control fluid flow and direct the arrangement of particles. This method induces particles, which can range from plastic microbeads to bacterial spores and even pollutants, to assemble and position themselves at a specified location within a liquid medium [109].

##### Drop Coating

A liquid droplet’s evaporation in its unsaturated vapor’s presence is a natural phenomenon. This process is exemplified by natural occurrences like the water cycle, which entails the condensation of water vapor and the evaporation of liquid droplets or other forms. When a droplet contains insoluble particles, it forms a deposition pattern on the solid surface following evaporation. The movement of these particles is inherently connected to the mode of droplet evaporation and the dynamics of the three-phase line [110].

Drop coating is a technique used to create thin layers or coatings on the surface of a sample or substrate. It involves carefully placing small droplets of a solution onto the surface and then allowing the solvent (the liquid part of the solution) to evaporate. As the solvent evaporates, it leaves behind a thin and uniform layer of the dissolved material, effectively coating the surface. This method is commonly used in various applications, including preparing thin films, protective coatings, and the deposition of materials onto surfaces. Drop coating is appreciated for its simplicity and effectiveness in creating precisely controlled, thin layers on a wide range of substrates [111,112].

**Figure 3:**
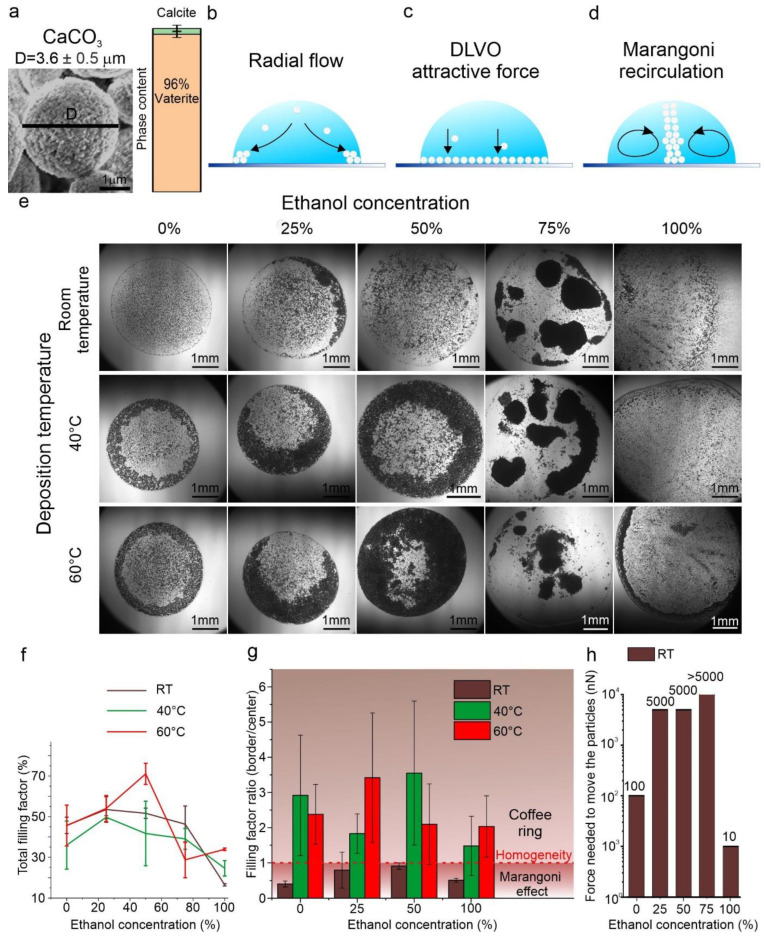
(**a**) SEM image of CaCO_3_ particles indicating the diameter of the particles (~3.6 micrometers) and weight fraction (%) of CaCO_3_ crystalline phases in XRD analysis. (**b**–**d**). Three flow schematics include radial flow, particle movement attracted to the substrates directed by the Derjaguin–Landau–Verwey–Overbeek (DLVO) force, and Marangoni flow. (**e**): the optical image of drops in different temperatures with different ethanol concentrations. (**f**) The filling factor of the whole surface area of the drop, which illustrates how close the coverage is to a monolayer. (**g**) The ratio of the filling factor of the border part to the central part, which corresponds to the particle distribution effects. (**h**) The force needed to move the particles drop coated on the substrate at room temperature using five different ethanol concentrations for stability check.

Eftekhari et al. [111] demonstrate that the balance between radial flow, the Marangoni effect, and the DLVO effect is the driving force to control the CaCO_3_ particle distribution (Figure 3a–d). For example, the process of achieving homogeneous CaCO_3_ particle deposition via drop coating is intricately linked to the control of the glass substrate’s roughness, as evidenced by two distinct types of substrate treatments. The first, piranha-treated glass, exhibited a very smooth surface with a contact angle of 0. In contrast, ethanol-treated glass, characterized by a roughness of approximately 0.3 nm, provided the optimal contact angle conducive for uniform particle deposition. The synthesized CaCO_3_ particles, measuring 3.6 ± 0.5 µm in diameter, demonstrated that homogeneous deposition on the substrate is feasible when the height of the drop (h) is greater than the particles’ diameter, a phenomenon clearly illustrated in Figure 3a.

Further investigation into the influence of temperature and ethanol concentration revealed that these variables critically affect particle distribution within a drop, as elaborated in Figure 3. This study uses the optical imaging analysis (Figure 3e) and filling factor as a metric (Figure 3f,g) to elucidate these effects. Notably, the relationship between the filling factor of the border and the central parts of the drop emerged as a pivotal factor in determining the deposition pattern.

A critical observation was the coffee ring effect, where an evaporating liquid leaves behind a ring-like deposit of particles. This effect was found to be contingent on the filling factor ratio, with values greater than 1 leading to the coffee ring phenomenon, as expounded in references [113,114] and illustrated in Figure 3g. Conversely, ratios less than 1 indicated the influence of the Marangoni effect, a flow within a fluid caused by variations in surface tension. The adhesion force between the particles and the substrate, quantified using Atomic Force Microscopy (AFM), varied with different ethanol concentrations in the solution (Figure 3h). This adhesion force directly influenced the friction between particles and the substrate, thereby affecting the stability of the particle deposition. This comprehensive analysis demonstrates the significant impact of substrate roughness, temperature, and ethanol concentration on the homogeneous deposition of CaCO_3_ particles via drop coating. The findings from this study, particularly in relation to the coffee ring effect and Marangoni flow, underscore the nuanced complexities of particle deposition dynamics and offer valuable insights for optimizing coating processes [111].

##### Microfluidic Devices

Microfluidic devices also leverage fluid flow for particle organization. These devices, which handle minute quantities of fluids using diminutive channels ranging in size from ten to hundreds of micrometers, can transport, mix, separate, or process fluids in other ways. They have found use in a variety of applications, including drug delivery systems, chemical sensors, and fluid pumps [110].

Compared to pure liquid droplets, droplets containing nanoparticles exhibit significant variations in spreading, evaporation, and liquid motion. These variations, induced by the nanoparticles, are primarily manifested in the static contact angle, contact line motion, evaporation rate, evaporation regimes, and flow dynamics within a drying droplet [115].

The recent attention to lipid nanoparticle (LNP) development using microfluidic devices, exemplified by applications, such as mRNA vaccines, is notable. For the production of lipid nanoparticles, two primary methods have been utilized: bulk mixing and microfluidic assembly. The latter is detailed in two studies focusing on the synthesis of LNPs using microfluidic devices with a mixer structure [116,117]. The microfluidic method, with its variable parameters, like flow rate and mixer structure, offers precise control over LNP size, which is crucial for applications in RNA therapeutics and vaccines, enhancing hepatic gene silencing and efficient intracellular delivery of siRNA and mRNA [116].

##### Capillary Assembly

Capillary assembly processes are highly specialized techniques employed in microscale and nanoscale assembly, where the adhesive properties of capillary forces are harnessed to precisely position and integrate miniature components [118]. These processes rely on the phenomenon of capillary action, wherein liquid is drawn into narrow channels due to surface tension, and it is used to manipulate and assemble tiny structures with remarkable precision [118]. Capillary assembly finds applications in various fields, including microelectronics, micro-electro-mechanical systems, and optics, enabling the creation of intricate devices and systems that benefit from the inherent accuracy and reliability of this assembly method. It plays a pivotal role in the miniaturization and advancement of technology across multiple industries [119].

#### 4.1.2. Dip Coating

Dip coating is a technique used to deposit a thin film or coating onto a substrate by immersing the object into a liquid solution containing the coating material and subsequently withdrawing it at a controlled rate. The dip coating process comprises five stages: Immersion, Start-up, Deposition, Drainage, and Evaporation. Various factors like submersion time, withdrawal speed, solution composition, concentration, and environmental conditions influence the final state of the dip coating, affecting the structure and thickness of the resultant film [120]. Dip coating is a robust option for a myriad of applications necessitating thin films or coatings deposition on substrates. It is simple, cost effective, and has the capability to produce uniform coatings using various materials, rendering it a favorable choice among manufacturers. Nonetheless, the challenges tied to controlling coating thickness and the potential slowdown in production due to drying and curing times may affect its suitability for certain applications [120].

This process finds application in manufacturing bulk products, like coated fabrics and specialized coatings in the biomedical field. It is also utilized in academic research for creating thin-film coatings. Dip coating is a low-cost process that does not necessitate expensive machinery, making it economical for both small-scale and large-scale production [120]. Another example of dip coating application is the creation of polymer films on brass wire substrates. Before dip coating, the wires were cleaned thoroughly, and a specific dip coater was utilized to make these coatings. This process was highlighted in a study to understand the thickness and structure of dip coated polymer films in a liquid state [121]. In an industrial setting, dip coating serves as a powder coating process, aiding in manufacturing high-volume products, like coated fabrics and specialized coatings, in the biomedical field. The process involves dipping a substrate into a coating material solution at a constant speed, making it a straightforward yet effective coating method. Sol-gel dip coating is a variant in which inorganic precursors are concentrated on the substrate surface through gravitational draining, evaporation, and condensation reactions. This process influences the structure of the deposited films, including factors like the size and opacity of fractal precursors [122].

#### 4.1.3. Spin Coating

**Figure 4:**
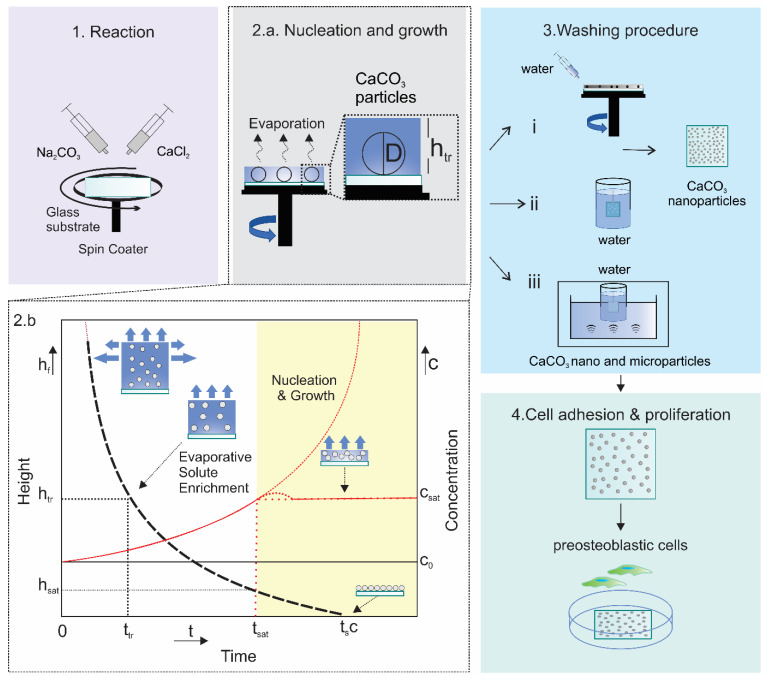
A schematic of the experiment, where step (1) is a reaction on a spin coater for the in situ synthesis of CaCO_3_. Step (2) 2.a: The nucleation and growth of CaCO_3_ particles during film thinning; 2.b: A schematic of the fundamental connections between film thinning (in blue), solute enrichment (black dashed line), nucleation, and growth (red dashed line) as they occur in the spin coating of a combination of a volatile solvent and a non-volatile solute adopted from [128]. The height at which the control of film thinning shifts from hydrodynamic forces to liquid evaporation is referred to as the transition height (h_tr_). When the film height (h_f_) is less than h_tr_ (at t_tr_), evaporation is the dominant process in film thinning. Until total process time at t = t_s_c, the film’s solute concentration, c, rises, and hf decreases simultaneously. This indicates that the saturation concentration of the solute, c_sat_, occurs at a film thickness of h_sat_ and a time of t_sat_. After t_sat_, the solute concentration will rise above c_sat_ until it breaks through the nucleation barrier and nucleation of the solute begins. Once the system is entirely dry, the solute concentration will drop to c_sat_ and stay at the same level. Step (3): Substrate washing with water after the spin coating process via three different procedures: (i) aliquot added on the rotating sample, (ii) dipping in water, or (iii) immersion in water with sonication. Step (4): The preosteoblastic cell adhesion and proliferation on the substrate coated with CaCO_3_ particles [124].

Spin coating is a widely used technique in semiconductor manufacturing, photolithography, and nanotechnology fields. It serves the purpose of applying thin films onto flat substrates. The process involves dispensing a small quantity of a liquid solution containing the desired material onto a spinning substrate (Figure 4). Through the combination of centrifugal force and solvent evaporation, the solution spreads evenly across the substrate, forming a thin and smooth film. The quality and thickness of this film depend on various factors, including the solution’s viscosity, the speed and duration of spinning, and environmental conditions [123,124].

This method, frequently employed in microfabrication, can produce films with thicknesses less than 10 nanometers. In photolithography, for instance, it is commonly used to deposit photoresist layers of approximately 1 micrometer thickness. Typically, the photoresist is spun at 20 to 80 revolutions per second for 30 to 60 s. Spin coating’s ability to create exceptionally thin layers has found applications in producing transparent titanium dioxide thin films on substrates like quartz or glass. These thin film coatings can possess self-cleaning and self-sterilizing properties [125].

The dynamic method constitutes a mathematical model essential for understanding the evolution of film thickness during the spin coating process. This model relies on the equilibrium between fundamental forces that act on the liquid film: centrifugal force, viscous force, and surface tension. It delineates the process into three sequential phases: deposition (the controlled dispensing of a specified volume of the solution onto the substrate), spin up (wherein the substrate accelerates to a constant angular velocity), and spin down (a phase where the substrate maintains a consistent speed until the film reaches a stable state) [126].

The dynamic method is a predictive tool for optimizing film thickness and uniformity across diverse material systems and substrate types. It facilitates a comprehensive understanding of how various parameters influence film formation and drying processes. Nevertheless, it is crucial to acknowledge its limitations stemming from specific assumptions. These assumptions include the neglect of solvent evaporation during the spin-up phase, the negligible influence of gravity, and the assumption of a flat substrate surface. Furthermore, this method fails to comprehensively address crucial factors, such as solvent diffusion, substrate surface roughness, and ambient humidity. Thus, the pursuit of ongoing research and development remains vital to augment the accuracy and applicability of the dynamic method [127].

For example, the innovative synthesis of CaCO_3_ particles [124], as depicted in Figure 4, involves a spin coating process where an aqueous solution of CaCl_2_ and Na_2_CO_3_ is added to a rotating substrate. This method ensures an even spread and mixture of the solutions, leading to the nucleation and growth of CaCO_3_ particles during film thinning.

The synthesis process hypothesizes three scenarios. (a) Nucleation occurs early in the hydrodynamic control stage, leading to a defined number of particles per unit volume (Figure 1, scenario a). (b) Creation and growth throughout the thinning process, resulting in a wide size distribution (scenario b). (c) Nucleation post-transition height (htr) due to solvent evaporation in low-concentration solutions, complicating the correlation between initial salt concentration and final particle coverage (scenario c).

The impact of precursor concentration and the washing procedure post-deposition is critical. At a spin coating speed of 1000 rpm and 35% relative humidity at 25 °C, variations in salt concentrations (from 0.03 mM to 1M) significantly influence particle size and distribution. Lower concentrations lead to nanoparticle formation post-htr, while higher concentrations result in microparticle deposition from the process’s beginning.

Three different washing procedures were tested to optimize the removal of excess precursors: in situ washing on the rotating plate, dipping the substrate in water, and immersing the substrate in water with sonication. The washing methods affected the final morphology and coverage of CaCO_3_ particles, with in situ washing showing inefficiency for removing excess salts. Such novel spin coating techniques and the study of varying parameters provide a nuanced understanding of the factors influencing CaCO_3_ particle distribution and size.

Karpitschka et al. [129] have developed a clear and practically useful theoretical approach for the evaporative spin casting of solutions with low solute concentrations, building on experimental data and previous research [130]. This analysis offers insights into the process of solute enrichment and film formation, enabling quantitative predictions of the spin casting process outcomes based on the processing parameters used [131]. For large particles, this basic theory fails. The causes of this failure were analyzed by Danglad-Flores et al. [127], and a corrected, more general theoretical approach was presented. It takes into account particle size effects as well as particle sedimentation [127].

#### 4.1.4. Other Dynamic Methods

As mentioned before, this review does not include all static and dynamic methods, as some, like the Langmuir–Blodgett (dynamic assembly) and freeze casting (dynamic assembly) assembly techniques, are less relevant to the primary focus of this study. Langmuir–Blodgett is a precise method for assembling monolayers of molecules into well-defined nanostructured films. It begins with spreading molecules at the gas–liquid interface and then transferring them onto a solid substrate through a vertical dipping process, which can be repeated to create multilayer structures [132,133]. This method has been instrumental in the controlled assembly of organic molecules for nearly a century, leading to advancements in surface science, nanotechnology, and functionalized surfaces [132,133,134].

Freeze casting is a technique for forming materials with high porosity and complex geometries. Studies have demonstrated its efficacy in shaping TiO_2_ and Al_2_O_3_ nanoparticle suspensions [135,136]. This method extends to fabricating nanocomposites by dispersing various nanoparticles in polymers, leading to innovative materials, such as MOF/polymer monoliths and magnetic filters with controlled porosity, which are achieved by freeze-drying dispersed nanoparticle solutions [137,138]. The process is highlighted for its precision in packing and pore directionality, which are essential for advanced material design.

### 4.2. Field-Driven Assembly

Field-driven active colloidal systems offer an interesting framework for achieving discrete dynamic functions crucial for building intelligent microscale devices. In these systems, particles dispersed in a fluid can be activated and directed by external fields, allowing them to perform actions like movement, interaction, assembly, and reconfiguration [101].

Magnetic and electric fields have emerged as powerful tools for remotely and precisely controlling a wide range of colloidal particles. This flexible control can be fine tuned by adjusting field parameters such as direction, strength, and frequency. Recent research has even shown that these field-driven active colloidal systems can replicate collective behaviors observed in living organisms, underscoring their potential in biomimetic applications [139].

When subjected to external fields, colloidal particles can exhibit diverse, dynamic behaviors, including acoustic, optical, magnetic, and electric fields. These energy sources have proven highly effective in producing collective patterns for several compelling reasons. Firstly, using magnetic and electric fields enables the remote delivery of energy over considerable distances, making it possible to simultaneously activate a large group of particles. Secondly, precise control over the activated particles can be attained by fine tuning field parameters, like field amplitude and frequency [140].

Particle dynamics, driven by magnetic and electric fields, often rely on generating an effective dipole within colloidal particles. When a magnetic or electric field is applied, these particles polarize and acquire an induced dipole moment. This induced dipole moment persists until the field is removed. The polarization of particles requires a disparity in polarizability between the particles and the surrounding medium. In the case of magnetic polarization, it is crucial to have magnetic materials present in either the particles or the medium. In contrast, electric polarization is less reliant on material selection due to the relatively modest difference in electric polarizability between most particles and an aqueous medium [141].

At the level of a single particle, external fields can apply torque to particles with an induced dipole. This occurs when the direction of the external field and the induced dipole within the particles do not align. As a result, the particles may experience field-driven torque and align themselves with the direction of the external field [142]. Consider, for example, a rotating magnetic or electric field. In this scenario, a magnetic or electric torque is present, causing the particles to rotate in sync with changes in the direction of the external field. In most instances, if the field direction remains constant, the particles will stay aligned along the field direction. This is because no external torque is present [143].

At the level of multiple particles, charged particles may display directed assembly patterns as directional particle–particle interactions come into play. Although these interactions have different origins, effective electric or magnetic polarization results in similar dipole–dipole (or dipolar) interactions. These interactions can be analyzed using the point–dipole approximation model [144]. A notable result of such field-induced dipolar interactions is dipolar chaining. This phenomenon occurs when polarized particles in a uniaxial field form linear chains of particles in a head-to-tail configuration. Crucially, these assembled clusters can generate non-uniform local fields of high intensity. These fields can attract neighboring particles through dielectrophoretic (or magnetophoretic) forces [141]. The combination of dipolar chaining and dielectrophoretic (or magnetophoretic) forces can lead to further assembly. Multiple linear chains can evolve into close-packed crystalline structures under the continuous application of the same field. This synergy results in complex and organized structures [141].

In time-dependent multiaxial fields, field-induced dipolar interactions can become more intricate. These fields involve periodic changes in the field’s direction and magnitude over time. When exposed to such time-dependent electromagnetic fields, polarized particles experience time-averaged dipolar interactions [145]. These interactions can result in the assembly of more complex structures than linear chains.

One straightforward example of a time-dependent field is an in-plane rotating field, which can be generated by combining orthogonally placed biaxial fields with two sinusoidal signals having a quadrature (90°) phase difference [146]. Additionally, a balanced triaxial magnetic field can be achieved by adding a *Z*-axis vertical field to an XY in-plane rotating field. These time-dependent multiaxial fields can potentially serve as a tool for assembling hierarchically ordered dynamic structures [147].

While magnetic field-driven assembly shows promise, it also faces specific challenges and limitations. One such challenge is that the magnetic force typically scales with the volume of the particles, which imposes a constraint on the miniaturization of structures [148]. Moreover, the magnetic force depends on the magnetic susceptibility of both the particles and the surrounding medium. This susceptibility can vary with factors like temperature or other environmental conditions, adding a layer of complexity to the process. Additionally, the magnetic force diminishes rapidly with distance, affecting the assembled structures’ stability and uniformity. Furthermore, there is a potential risk of the magnetic field interfering with other devices or systems reliant on electromagnetic signals, which could pose significant issues in certain contexts [149].

Another approach involves the simultaneous use of multiple field stimuli. This approach could introduce a higher level of ordering in particle assemblies [141]. Given their distinct origins, magnetic and electric dipoles operate independently. This principle was demonstrated by Bharti, Velev, and their colleagues. They showcased the directed assembly of multi-directionally percolated network structures in concurrent electric and magnetic fields [141]. In addition to combining electric and magnetic fields, integrating other fields, such as optical fields, is possible. This integration can offer further control over assembled structures’ spatial and temporal organization. Furthermore, using complex particles, such as building blocks, extends beyond simple spherical particles and includes particles with anisotropic shapes and/or polarizability [150]. Employing these complex building blocks can result in more intricate multipolar interactions, ultimately leading to assemblies characterized by a broad spectrum of structural diversity [151].

Gravitational fields, a distinctive form of field-driven assembly, arise from the mass distribution of the particles themselves rather than an external source. These fields can either attract or repel, depending on the relative orientation and shape of the particles. They provide a means for crafting innovative structures and materials with customizable properties, such as density, stiffness, permeability, or optical response [152].

For instance, consider magnetic field-driven particle assembly and jamming, which can yield a material with dynamically adjustable magnetic permeability or dielectric permittivity. However, gravitational fields also pose challenges for field-driven assembly due to their potential to induce divergence or instability in particle configurations. Accurately modeling and measuring the gravitational field of an irregularly shaped mass body, such as an asteroid or comet, can be particularly daunting [153].

#### 4.2.1. Electromagnetic Field

Optical tweezers, also known as optical traps, are scientific instruments that employ tightly focused laser beams to manipulate extremely small entities, including nanoparticles [154], droplets, and biological cells [155,156]. The laser’s radiant energy exerts either an attractive or repulsive force, typically at the picoNewton level, depending on the refractive index difference between the particle and its surrounding medium [157]. These optical devices leverage laser power to precisely control micron and sub-micron scale matter, enabling intricate scientific investigations and experimental manipulations. They play a crucial role in elucidating interactions and forces governing particles at the microscopic level, advancing the understanding of fundamental physics and biological systems [158].

The operating principle of optical tweezers is based on a fundamental optical phenomenon: the momentum carried by light, which is proportional to its energy and propagation vector [159]. As a laser beam passes through an object, it undergoes refraction, altering its momentum. According to Newton’s third law, the object experiences an equal and opposite shift in momentum, creating a reaction force necessary for maintaining overall momentum equilibrium [157]. Optical tweezers are particularly adept at capturing dielectric particles and find applications in biology, pharmacology, and clinical research [160]. They enable precise manipulation of molecules, offering insights into molecular dynamics, force quantification during stretching, and studying structural transitions within biomolecules [160].

In 2018, Ashkin received the Nobel Prize in Physics for his pioneering work on optical tweezers, highlighting the groundbreaking nature of his contributions [161]. His innovations have revolutionized various scientific fields, allowing the exploration of biomolecules at the single-molecule level and providing unprecedented insights into biological systems [108].

**Figure 5:**
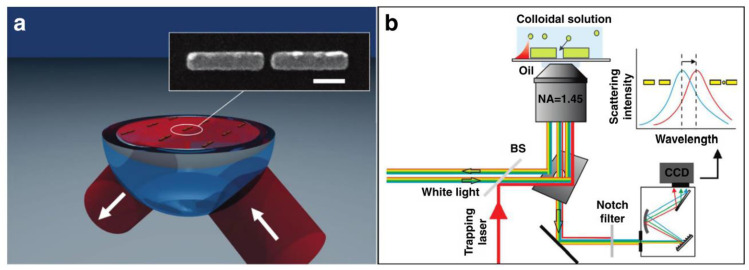
Principle of structural plasmonic tweezers. (**a**) Surface plasmonic trapping configuration through the design of nanostructures; scale bar: 200 nm [164]. (**b**) Experimental configuration. The trapping events can be directly monitored using the scattering spectra of the antennas [165].

Controlling and manipulating microscopic entities in a fluidic environment is paramount in numerous domains, including physical and chemical analysis, diagnostics, medicine, food processing, and environmental monitoring [162]. Plasmonic tweezers (Figure 5), with their ability to create engineered plasmonic hotspots through structural design or laser beam characteristics modulation, offer significant advantages in terms of near-field energy generation and precise manipulation [163]. These platforms hold immense potential for particle transport, sorting, and segregation within liquid conduits, paving the way for new possibilities in scientific research and technological advancement [161].

While challenges persist in plasmonic tweezers, ongoing exploration and innovation are essential to overcome inherent limitations [166]. The future promises a growing landscape of applications, ushering in novel frontiers and expanding the horizons of scientific inquiry and technological progress [167].

#### 4.2.2. Electric Field

Electrokinetic assembly is a captivating scientific technique that utilizes the potent influence of electric fields to meticulously organize charged entities, including nanoparticles, microspheres, and biological cells, on a substrate. This versatile method enables the construction of diverse structures in terms of shapes, dimensions, and functional attributes [156]. The specific outcome hinges on three crucial factors: the magnitude of the applied voltage, the electric field frequency, and the inherent properties of the particles involved. Electrokinetic assembly is a significant tool in nanotechnology, biotechnology, and materials science, with applications extending from advanced plasmonic devices to highly sensitive biosensors and innovative tissue engineering [168]. The interplay between electric forces, particle characteristics, and external variables in electrokinetic assembly opens an exciting avenue for researchers, promising innovative technologies that could revolutionize the fields of science and engineering.

The principles underpinning electrokinetic assembly are akin to those of a battery. Electrodes, comprising a cathode and anode, are introduced, and charged, facilitating the mobilization of particles, such as ions, through the electric current [136]. Ions and water migrate toward the electrodes. Each electrode assembly consists of water, a pump, and an electrode. Describing an electrokinetic system necessitates governing equations for local bulk fluid velocity, local species concentrations, and the mean electrical potential (φ) [169].

Electrokinetic assembly, however, unveils a complex landscape filled with challenges. Recent investigations have called into question long-standing assumptions, casting a shadow of uncertainty on conventional wisdom in this field [170]. The remediation of sites tainted with heavy metals, particularly those compounded by mixed contaminants, such as organic compounds, intertwined with heavy metals and/or radionuclides, presents a formidable challenge [171]. These sites, mired in environmental contamination, defy straightforward solutions, necessitating a comprehensive exploration of advanced strategies and technologies to mitigate complex contamination profiles and potentially restore environmental integrity [172].

Despite these formidable obstacles, recent advancements in electrokinetic assembly offer promising opportunities for crafting intricate nanostructures. A prime example is the pioneering technique of orchestrating gold nanostructures tailored for surface-enhanced Raman scattering applications [173]. This innovative approach employs alternating current electrokinetic forces to manipulate gold nanoparticles precisely, guiding them toward assembling two distinct structures. These structures, nanowires, and elaborate branched configurations resembling “nanotrees” exhibit nuanced dependencies on frequency. This expansion of available structures holds tremendous promise across diverse scientific domains, including optics, electronics, and magnetics, heralding transformative advancements that could reshape the landscape of research and technological innovation [174].

Guided electrokinetic assembly, which amalgamates dielectrophoresis and electro-osmosis, offers a strategic fusion of self- and direct-assembly techniques [175]. This orchestrated approach operates on a scale suitable for assembling minuscule components and employs a non-contact methodology for component positioning, achieving a commendable throughput akin to self-assembly techniques [175]. Dielectrophoresis and electro-osmosis, integral components of guided electrokinetic assembly, have found extensive adoption in diverse domains, facilitating the intricate tasks of transporting, sorting, separating, and assembling nano- and microparticles and biological cells [176]. Nevertheless, the effectiveness of electrokinetic forces may occasionally encounter challenges stemming from elusive and multifaceted influences, including thermal forces, fluid viscosity, particle interactions, and other factors that are difficult to quantify. As a result, the precision of particle placement using electrokinetic forces may sometimes fall short of the accuracy achievable through sophisticated pick-and-place systems commonly employed in industrial settings [177].

#### 4.2.3. Magnetic Field

Magnetic tweezers (MTs) are a revolutionary scientific instrument with remarkable capabilities within biophysics and nanotechnology. This versatile apparatus is a potent asset, facilitating the manipulation and scrutiny of biomolecules and polymers with unparalleled precision and finesse [178]. Operating at the intersection of forces and molecular entities, MTs allow for meticulous control over spatial dynamics and positioning of individual molecules and molecular clusters [101]. This level of control serves as a conduit to unlocking the intricate details governing these entities’ behavior and structural configurations. The utility of MTs extends beyond mere manipulation; it serves as a portal to unveil the profound secrets hidden within the molecular world, offering insights that resonate across diverse scientific and technological frontiers [178].

In biophysics, magnetic tweezers have emerged as an invaluable single-molecule methodology. They empower scientists to explore the mechanical characteristics of nucleic acids and elucidate the intricate interactions between proteins and nucleic acids in real time, all at the level of individual molecules [179]. This exquisite technique serves as a gateway to gaining profound insights into the biomechanical underpinnings of biological entities, unraveling the elusive tapestry of their structures and functions. In nanotechnology, MTs play a pivotal role as a virtuoso conductor, orchestrating the assembly and manipulation of magnetic nanoparticles with precision [180]. These nanoparticles are ingeniously engineered with tailored characteristics, guided by magnetic fields to perform intricate choreography, culminating in the construction of intricate nanoscale architectures, like magnetic nanochains or nanocomposites.

The extensive capabilities of MTs open the door to many potential applications that reverberate across scientific landscapes. From data storage, where MTs could be utilized to manipulate magnetic bits at the nanoscale, and environmental remediation, where MTs could assist in the precise manipulation of magnetic nanoparticles for contaminant capture, to the frontiers of novel materials development, MTs’ impact promises transformative outcomes poised to redefine both technological and scientific horizons [181].

Magnetic tweezers rely on the principles of electromagnetism, using precisely shaped pole tips to establish a magnetic field gradient. These instruments encompass various categories, each distinguished by the number of magnetic poles harnessed [182]. Single-pole magnetic tweezers provide a straightforward but limited approach, generating attractive forces directed toward the pole tip. Seeking greater degrees of freedom in magnetic force application, researchers have pioneered multipolar magnetic tweezers, resulting in ingenious setups [26]. For instance, Harber and Wirtz introduced a two-pole configuration, orchestrating back-and-forth forces through the interplay of opposing poles [183]. De Vries et al. ventured into 2D force manipulation, crafting a micromagnetic manipulator adorned with three in-plane poles [184]. Amblard et al. presented an eight-pole apparatus to amplify spatial dynamics by orchestrating four coils, offering intricate 2D lateral movements and rotations [185]. Grosse and Croquette expanded their horizons with their six-pole instrument, proficient in generating magnetic forces spanning the vertical and near-horizontal dimensions [186]. Fisher et al. unveiled a hexapole design, representing the apex of magnetic prowess, capable of initiating magnetic forces in an arbitrary 3D expanse [187]. This rich tapestry of multipolar magnetic tweezers continues to evolve, providing a transformative perspective into the intricate cosmos of magnetic manipulation.

The foundational concept of magnetic manipulation can be traced to the alignment of iron filings in the presence of a magnet, akin to the approach employed by Crick and Hughes. They demonstrated the first use of magnetic actuation to manipulate magnetic particles within the cytoplasm of cells, pioneering the magnetic tweezers technique [188]. Subsequent research by Smith et al. and Strick et al. expanded on this technique, conducting elegant experiments in which magnetic actuation was employed to stretch and coil individual DNA molecules tethered between a flow cell surface and microscopic magnetic particles [189]. These contributions laid the groundwork for what is now widely recognized as single-molecule magnetic tweezers [190].

Within the intricate realm of magnetophoretic devices in microchannels, particles experience a symphony of forces orchestrating their dynamic journey. These forces include the magnetic, drag, gravitational, buoyant, and lift forces, each playing an important role in the multidimensional theater of particle motion [191]. This intricate ballet, governed by the fundamental principles encapsulated in Newton’s second law, harmonizes many forces in a delicate equilibrium, presiding over the trajectory of particles or cells traversing the microfluidic domain. This physical edict is elegantly expressed in the following equation:

(1) $$m\frac{d_{up}}{d_{t}}=F_{m}+F_{d}+F_{g}+F_{b}+F_{L}$$

Here, *m* (in kilograms) stands as the solitary particle or cell’s mass, *d_up_* (in meters per second) denotes its velocity, and d_t_ is time in seconds. At the same time, *F_m_* (in newtons) symbolizes the magnetic force, *F_d_* (in newtons) embodies the hydrodynamic drag force, *F_g_* (in newtons) signifies gravitational force, *F_b_* (in newtons) encapsulates buoyancy force, and *F_L_* (in newtons) represents the lift force [189].

In microfluidics, this intricate interplay of forces, as depicted in the mathematical expression, serves as the guiding compass for understanding the choreography of particle dynamics. It is a tapestry of physical phenomena that elucidates the nuanced intricacies of microparticles and cell behavior, opening gateways to profound scientific exploration [192].

Magnetism in materials arises from the intrinsic spin of electrons and their orbital motion around the atomic nucleus. This phenomenon categorizes materials into three classes based on their responses to magnetic fields: diamagnetic, paramagnetic, and ferromagnetic materials, each exhibiting a unique magnetic character [189]. Diamagnetic materials, including substances like water, wood, and most biological cells, possess inherent non-magnetic properties. When exposed to an external magnetic field, the atomic constituents within diamagnetic materials experience a subtle disruption in the equilibrium of orbiting electrons, inducing the creation of minuscule magnetic dipoles within the atoms [193]. Importantly, these dipoles manifest with orientations that steadfastly oppose the direction of the applied magnetic field. On the other hand, paramagnetic materials exhibit a relatively weak attraction when exposed to a magnetic field. Without such a field, the magnetic dipoles inherent to paramagnetic materials assume random orientations. However, the introduction of an external magnetic field imparts upon these materials a detectable magnetic susceptibility, resulting in a feeble but discernible magnetic attraction [194].

Microfluidic devices often leverage various sources of magnetism to generate the necessary magnetic fields and gradients crucial for their functionality. A primary choice in this regard is neodymium–iron–boron (NdFeB) permanent magnets, renowned for their effectiveness. These magnets, available in diverse shapes and configurations, allow strategic placement near microchannels to create specific magnetic fields, typically ranging from 0.5 to 1 teslas, with field gradients spanning hundreds of teslas per meter. These magnetic fields are essential for precisely manipulating cells and microparticles within the microfluidic environment. One notable advantage of NdFeB permanent magnets is their self-sufficiency, as they do not rely on external power sources, simplifying their setup and use [195]. Additionally, integrating high-gradient microscale permanent magnets directly onto microfluidic chips near microchannels is a practical and advantageous approach [196].

#### 4.2.4. Acoustic Field

Acoustic assembly provides a method for the rapid parallel fabrication of objects directly from a solution. In this process, particles suspended in a fluid accumulate in regions of high pressure under the influence of acoustic radiation forces. A UV-triggered reaction is initiated to stabilize the assembled structures [197]. Before this, the particles undergo a preparatory phase where a photoinitiator is loaded to sensitize the reaction, achieved through solvent-induced swelling [126]. This localized approach for photoinitiation confines the reaction to specific regions, leaving the overarching suspension available for subsequent use, thus ensuring efficiency and resource conservation. The resulting structures exhibit mechanical stability and self-sustaining properties, marking the successful fabrication of functional objects with precision and reliability [198].

Sound propagation, through either compression or shear waves, fundamentally depends on the medium’s mechanical properties [199]. When a particle interacts with its surrounding medium, a discrepancy in these mechanical properties leads to a phenomenon known as scattering, which redirects a portion of the sound wave’s momentum, generating a radiation force [128]. This force propels suspended particles within the fluid medium along migratory paths [198]. The direction of this migration is governed by several factors, primarily the material properties in conjunction with the acoustic contrast [200]. These parameters determine whether particles are driven toward regions characterized by high pressure or away from such zones [200].

Acoustic contrast, particularly applicable to particles considerably smaller than the acoustic wavelength, is defined by the ratios of mechanical compressibility and densities of both the surrounding medium and the particle [177]. Additionally, particle size is a crucial determinant, as larger particles may exhibit resonance at specific frequencies, significantly amplifying the radiation force or even reversing its direction [177]. Absent these effects, rigid and dense particles, relative to their environment, migrate toward low-pressure domains, while softer and lighter particles move along pressure gradients toward high-pressure regions [201]. In scenarios with standing waves, this migration leads particles to nodes or antinodes in the latter scenario [201].

Poly(dimethylsiloxane) (PDMS) microspheres were employed, a silicone rubber widely utilized across various scientific domains [126]. This choice is driven by PDMS’s notable attributes, such as biocompatibility, robust mechanical properties, and chemical stability, although its relatively inert chemical reactivity is acknowledged [197].

Acoustic waves, highlighted through the medium of acoustic tweezers, possess a range of capabilities extending beyond mere manipulation of individual entities [202]. This exploration delves into the extensive influence of sound waves, covering particle and cell sorting, polymer substrate fabrication, and cell differentiation [202]. The potential of acoustic stimulation in high-throughput applications in pharmaceutical sciences and in vitro tissue engineering is also discussed [203]. However, the technology is still in its early stages of development [203].

A significant area warranting further investigation lies in understanding the long-term effects of acoustic stimulation, especially concerning cell lineage commitment among stem cells [125]. The domain of regenerative medicine also invites exploration to uncover the full potential of acoustic waves [204]. Discerning the individual parameters that dictate cell fate is a complex challenge, like many scientific techniques. This complex interplay constitutes a frontier for further research to unearth scientific insights and innovations [177].

The complex dynamics surrounding cell proliferation and differentiation changes within hydrogels or cell culture media, whether attributed to shear stress induced by cell movement or other mechanical factors, temperature variations, or other parameters, remain a complex puzzle [205]. Likely, a synergy of multiple factors operating at specific critical thresholds directs the outcomes toward positive or negative effects [199]. A thorough examination of these dynamics necessitates extensive parameter studies [199]. Delving into gene expression changes presents an intriguing avenue for investigation [177]. Sound waves, applied precisely, emerge as a potential tool to selectively modulate cellular phenotypes, thus unveiling the underlying mechanisms governing genetic alterations [177].

Expanding consideration to encompass cellular dynamics and the kinetics and mechanisms governing gelation influences the long-term success of acoustic manipulation [201]. The process of hydrogel crosslinking, intertwined with the concurrent entrainment of cells, introduces shifts in hydrodynamic forces [201]. These forces, in turn, significantly influence the trajectory of cell fate, adding layers of complexity to the scientific endeavor [201].

## 5. Advantages and Limitations

As mentioned above, nanoparticle self-assembly is a process driven by particle interactions, with the goal of reaching thermodynamic equilibrium and minimizing the system’s free energy. In static self-assembly, interactions among nanoparticles lead to a minimum free-energy state. On the other hand, a dynamic system is maintained away from equilibrium by continuously supplying the system with an external energy source, which balances attractive and repulsive forces [206]. Colloidal crystals (CCs) that self-assemble are promising candidates for Photonic Crystals (PhCs). They have opened up unparalleled opportunities in various fields, including photonics, optics, optoelectronics, sensing, energy harvesting, environmental remediation, pigments, and more [207]. The potential for development in the self-assembly of nanoparticles is immense. This pivotal method is instrumental in the fabrication of functional materials and devices. The technique involves non-contact manipulation, eliminating the need for auxiliary substances and thereby preventing unwanted contamination. Moreover, this process can be carried out continuously, ensuring the preservation of the particles’ original attributes [208].

Dynamic methods of particle assembly (DMPA) are sophisticated techniques that enable the manipulation and organization of particles into specific configurations. The precision offered by dynamic methods of particle assembly is particularly advantageous in fields such as nanotechnology, where the spatial arrangement of particles can drastically alter the characteristics of the resulting material [209]. Versatility is another strength of dynamic methods, as it can handle particles with diverse physical properties, broadening its applicability across various domains. Furthermore, certain DMPA methods, like acoustic tweezers, are non-invasive, ensuring no harm to cells during manipulation. The extensive range of acoustic frequencies used by these tweezers allows for the handling of particles of sizes spanning from nanometers to millimeters [210]. Implementing dynamic methods of particle assembly can be intricate, necessitating a profound comprehension of the foundational principles for efficacious utilization. Certain DMPA techniques may entail substantial equipment expenditures. The assembly’s precision can be swayed by the distribution model employed. A few DMPA methods might offer a low resolution, potentially curtailing their efficacy in specific applications. It is crucial to underscore that the pros and cons can fluctuate based on the particular particle assembly method utilized and the context of its application [211].

One of the primary challenges in utilizing particle manipulation in biomedical research is the absence of a straightforward, operable method for the on-demand engineering of longitudinal dynamic self-assembly of particles. To address this, a microfluidic system induced by viscoelasticity is proposed [212]. This system aims to enhance the maneuverability and orderliness of the longitudinal dynamic self-assembly of particles and achieve on-demand control of interparticle spacings and the frequency of particles passing through an outlet [213].

Dynamic methods of particle assembly find broad applications in a variety of fields. These encompass areas such as biomedical research, nanotechnology, photonics, optoelectronics, and materials science. The extensive applicability of dynamic methods of particle assembly highlights its versatility and potential [214].

Dynamic methods for developing targeted drug delivery systems utilize a variety of carriers and strategies. The aim is to ensure the delivery of drugs to specific tissues, cells, or even intracellular organelles. Nanocarriers, including nanotubes, nanowires, nanoshells, quantum dots, nanopores, gold nanoparticles, dendrimers, noisomes, ufasomes, virosomes, cubosomes, nanobots, and transferosomes, are employed for drug targeting. These carriers can be manipulated to transport and release drugs at the intended site of action [215].

External stimuli, such as magnetic fields and ultrasound, can be used to perform imaging, targeting, and releasing drugs from the nanocarriers at the intended site of action. This approach allows for more controlled and precise drug delivery. Surface-functionalized nanocarriers can enhance the presence of the active therapeutic substance at specifically desired locations in the body, thereby minimizing nonspecific side effects. This method improves the specificity of the system toward the pharmacologically relevant target in the body [216]. Prodrugs, which are biologically inactive compounds, can be metabolized in the body to produce the active drug. They can be used to improve the drug’s physicochemical properties, such as solubility and stability, and to reduce its toxicity [217].

Dynamic methods of creating targeted drug delivery systems offer a promising approach to improving the efficacy and safety of drug treatments. However, further research is needed to overcome the challenges associated with these methods and to fully realize their potential in clinical applications [218].

## 6. Nanoarchitectonics in Biomaterial Sciences

Nanoarchitectonics is a technological approach that enables the organization of nano-sized structural units, typically groups of atoms or molecules, into a specific arrangement. It encompasses two primary processes: nano-creation and nano-organization. Nano-creation pertains to the synthesis of novel materials that are not found in nature, while nano-organization involves the rearrangement of these structural units into a desired pattern [219].

This method extends beyond merely creating and organizing materials at the nanoscale. It also includes understanding and harnessing the ultimate functions of these materials. This is accomplished through various actions such as atomic- and molecular-level manipulation, chemical reactions, self-assembly, and self-organization, and their modulation by external fields and/or stimuli [10].

By controlling the arrangement of these nanoscale materials, nanoarchitectonics can effectively manipulate the properties of the resulting material systems [220,221]. This renders it a potent tool for the fabrication of functional materials with tailored properties for a variety of applications [124,222], for example, as a cell adhesion template [124,223].

**Figure 6:**
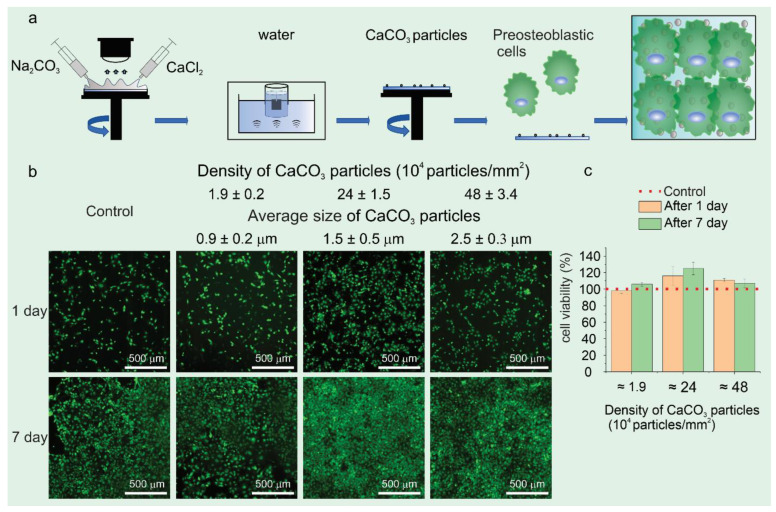
(**a**) Description of the preosteoblastic cells study on the CaCO_3_-deposited glass. (**b**) MC3T3-E1 cells cultivated on the surface of the non-coated glass and CaCO_3_-coated glass with three different particle coverages for 1 and 7 days. (**c**) Cell viability was measured at respective time intervals using an alamarBlue test on MC3T3-E1 cells seeded on CaCO_3_-coated glasses in the culture medium [124].

The effects of CaCO_3_ particles on preosteoblastic cell adhesion and proliferation were examined using MC3T3-E1 cells on non-coated and CaCO_3_-coated glass surfaces. Different synthesis parameters stimulate the various particle coverage and various particle sizes. Particle coverages of 1.9 ± 0.2 (10^4^ particles/mm^2^), 24 ± 1.5 (10^4^ particles/mm^2^), and 48 ± 3.4 (10^4^ particles/mm^2^) with respective sizes of 0.9 ± 0.2 µm, 1.5 ± 0.5 µm, and 2.5 ± 0.3 µm were studied over one and seven days (Figure 6a) via fluorescence microscopy (Figure 6b). This indicated that osteoblasts adhered well to CaCO_3_-coated surfaces, especially at a coverage of 24 ± 1.5 (10^4^ particles/mm^2^). Cell viability tests showed no significant toxicity from the CaCO_3_ particles (Figure 6c). A strong correlation was observed between cell and particle coverage via fluorescence microscopy, with results confirming that cell coverage increased notably with particle coverage up to 24 ± 1.5 (10^4^ particles/mm^2^) but not significantly beyond that.

After seven days, cell coverage was highest on surfaces with a particle coverage of 24 ± 1.5 (10^4^ particles/mm^2^). This suggests an optimal range for particle density to enhance cell adhesion and proliferation.

Furthermore, the study found that CaCO_3_ coating increases hydrophilicity and alters the electrostatic properties of the glass surface, influencing cell adhesion. The negative zeta potential of bare CaCO_3_ particles (−(26 ± 5) mV for 430 nm and −(12.2 ± 2.5) mV for 3 µm) and the two-step process involving electrostatic forces and integrin assembly were crucial in determining cell adhesion and proliferation. As a result, the study concludes that CaCO_3_ particles enhance cell adhesion and proliferation on glass surfaces, with a coverage of 24 ± 1.5 (10^4^ particles/mm^2^) providing the most favorable conditions.

### 6.1. Nanoarchitectonics for Drug Delivery

Liposomes, dendrimers, and polymer nanoparticles [224] are all nanoscale structures that have found significant use in various fields, including medicine, due to their unique properties. Liposomes are small, artificial vesicles that are spherical in shape and have at least one lipid bilayer. They are composite structures made of phospholipids and may contain small amounts of other molecules [225]. Due to their hydrophobicity and/or hydrophilicity, biocompatibility, particle size, and many other properties, liposomes can be used as drug delivery vehicles for the administration of pharmaceutical drugs and nutrients [226].

Dendrimers are highly ordered, branched polymeric molecules. They are typically symmetric about the core and often adopt a spherical three-dimensional morphology. Dendrimers are characterized by special features that make them promising candidates for a lot of applications. They are highly defined artificial macromolecules, which are characterized by a combination of a high number of functional groups and a compact molecular structure [227].

Polymer nanoparticles are a form of nanocomposite, comprising a polymer or copolymer with nanoparticles or nanofillers dispersed within the polymer matrix. The shift from micro- to nanoparticles results in alterations of their physical and chemical properties. These nanoparticles find extensive applications across various fields, including medicine, where they are utilized for targeted drug delivery [228]. Each of these nanoscale structures possesses unique properties and uses. Their diminutive size and large surface area make them ideal for applications necessitating precision and control at the nanoscale. However, further research is required to fully comprehend their potential and limitations [229].

**Figure 7:**
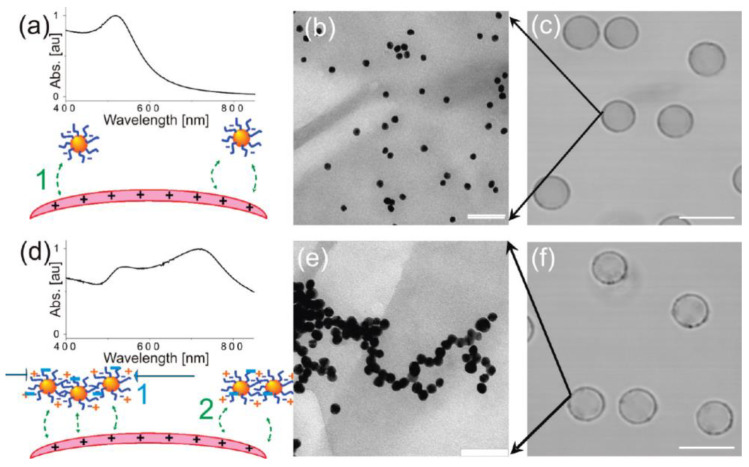
Distribution control of pre-synthesized gold NPs by adsorption onto polyelectrolyte multilayer capsules in the low concentration limit. (**a**) The absorption spectrum and the scheme of interaction for non-aggregated NPs (non-aggregates are obtained upon direct adsorption by admixing (1 in the scheme)); (**b**) TEM image of non-aggregated NPs on a microcapsule; (**c**) CLSM image of the microcapsules; (**d**) absorption spectrum and the scheme of interaction for aggregated NPs (aggregates are obtained by aggregating NPs first (1 in the scheme) and then adsorption (2 in the scheme); (**e**) TEM image of aggregated NPs on a microcapsule; (**f**) optical image of the microcapsules. The scale bars in b and e correspond to 100 nm; those in c and f correspond to 6 µm [231].

Also, the assembly of noble metal nanoparticles (NPs) in capsule shells could provide additional sensitivity for laser irradiation, leading to a more effective drug release [230]. Furthermore, the 2D and 3D assembly of the nanoparticles in the structure could affect the plasmon resonance adsorption peak and, as a result, increase the sensitivity to laser irradiation (Figure 7) [231,232,233].

### 6.2. Nanoarchitectonics for Tissue Engineering

Scaffolds with controlled porosity and surface chemistry are pivotal in various fields, especially in tissue engineering and regenerative medicine. The porosity of a scaffold is a key parameter as it influences cell seeding, proliferation, and the transport of nutrients and metabolic waste. Techniques such as 3D printing and phase inversion/salt leaching have been employed to fabricate scaffolds with controlled porosity [234]. For example, 3D printing can be utilized to create fully bio-based porous scaffolds with a specific porosity and pore size. Similarly, the phase inversion/salt leaching technique can regulate scaffold porosity features by modifying the amount and size of the porogen agent used [235].

The surface chemistry of a scaffold can significantly impact cell attachment, proliferation, and differentiation. Techniques such as the covalent attachment of cell adhesion-mediating peptides to the hydrophilic fibers of a scaffold can foster specific bioactivation. This enables cells to adhere through the exclusive recognition of the immobilized binding motifs [236]. This strategy allows synthetic materials to directly regulate cell behavior. The capacity to control both the porosity and surface chemistry of scaffolds paves the way for the design of advanced biomaterials. These scaffolds can mimic the extracellular matrix, providing a three-dimensional template that supports temporary loads and guides the growth of tissue to achieve its final form [237].

### 6.3. Nanoarchitectonics for Biosensors

Sensors featuring nanoscale characteristics have seen a surge in usage for biomolecule detection, including glucose, due to their high sensitivity and specificity. One such method is nanopore sensing, which operates on the principle of measuring ionic current variations as charged biomolecules, immersed in an electrolyte and translocated through nanometer-sized channels in response to an externally applied voltage across the membrane [238]. This technique enables the detection and characterization of individual biomolecules. The fabrication of nanobiosensors involves the use of various nanomaterials, including nanoparticles, nanowires, nanorods, carbon nanotubes, and quantum dots. These nanomaterials offer several advantages such as high stability, high carrier capacity, large surface area, high electrical and thermal conductivity, and color tunability [239].

Glucose sensors, such as the Dexcom G7 and the FreeStyle Libre, employ nanoscale features to monitor glucose levels continuously. These sensors provide real-time glucose readings to your smartphone, eliminating the need for regular finger pricks. They are designed to assist diabetes patients in easily tracking their blood glucose levels [240]. Sensors with nanoscale features present a promising approach for biomolecule detection. Their diminutive size and large surface area make them ideal for applications requiring precision and control at the nanoscale. However, further research is necessary to fully comprehend their potential and limitations [241].

### 6.4. Nanoarchitectonics for Imaging and Diagnostics

Contrast agents are substances utilized in medical imaging to amplify the contrast of structures or fluids within the body. They have the ability to absorb or modify external electromagnetism or ultrasound, which distinguishes them from radiopharmaceuticals that emit radiation themselves [242]. In the context of X-ray imaging, contrast agents enhance the radiodensity in a target tissue or structure. One of the enhanced properties of contrast agents is their water solubility. They are stable under heat, chemical reactions, and storage conditions. These agents are non-antigenic, meaning they do not trigger an immune response. They possess the right viscosity and density, and their low viscosity makes them easy to administer. They are persistent enough in the area of interest to allow its visualization and are selectively excreted by the patient once the examination is complete. Furthermore, they have the same osmolarity as plasma or lower, which is beneficial for patient safety [243].

Gold nanoparticles (AuNPs) serve as an exemplary instance of contrast. They are particularly attractive for biomedical studies, owing to their unique optical properties. The versatile optical properties of various gold nanostructures can significantly enhance the performance of biosensing and biomedical imaging. This results in improvements in sensitivity, specificity, speed, contrast, resolution, and penetration depth [244]. For example, conjugates of aggregated, photosensitized gold nanoparticles can be utilized for multimodal imaging and synergistic phototherapy. This allows for the effective destruction of cancer cells at power densities below the skin tolerance threshold, marking a significant advancement in the field of medical imaging and therapy. Contrast agents with enhanced properties, such as gold nanoparticles, hold promising potential in enhancing the quality and effectiveness of medical imaging. However, further research is necessary to fully comprehend their potential and limitations [245].

## 7. Comparative Analysis of Static and Dynamic Methods: Challenges and Future Perspectives

Biomaterial sciences have seen the rise of nanoarchitectonics as a potent strategy for crafting functional material systems. This method harmonizes a range of actions. These include manipulation at the atomic or molecular level, chemical reactions, and self-assembly and self-organization. Additionally, these actions are modulated by external fields or stimuli. The foundational principles and target scales of nanoarchitectonics mirror those of biological systems. In these systems, every facet of life relies heavily on physicochemical events occurring at the nano- to microscale [61].

In nanoarchitectonics, particle assembly is a key process, with both static and dynamic methodologies playing pivotal roles. Static methods, characterized by their time-invariant processes, are typically employed in the formation of particles into fixed patterns. Conversely, dynamic methods, which are time-dependent, are utilized in the self-assembly of particles under specific conditions [61]. The effectiveness of these two methodologies in particle assembly can be evaluated based on a variety of factors. For example, static methods may be more advantageous in creating stable structures with a high degree of order. In contrast, dynamic methods may prove more beneficial in the formation of structures capable of adapting to fluctuating conditions. The scalability of these methodologies in particle assembly can also be compared [15]. Similar to the aforementioned efficiency comparison, static methods may be more appropriate for the generation of stable, highly ordered structures. On the other hand, dynamic methods may be more suitable for the development of adaptable structures. The precision and control offered by these methodologies in particle assembly can be assessed as well [53]. Once again, static methods may be more fitting for the production of stable, orderly structures, while dynamic methods may be more effective for the construction of adaptable structures. Lastly, the compatibility of these methodologies with biological materials in particle assembly can be compared. As with the previous comparisons, static methods may be more apt for the creation of stable, orderly structures, whereas dynamic methods may be more efficient for the formation of adaptable structures [64].

Table 1 provides the advantages and disadvantages of each assembly technique studied in this review. Static methods like layer-by-layer assembly [51,52,53,58,59,60,61,62] and template-assisted assembly [2,10,16,29,70,72,73,74,75] may need specific substrates. Polymer brushes [83,84,85,86] offer adjustability but risk aggregation. Among dynamic methods, directed assembly and fluidic assembly provide versatility but require fine tuning [109]. Drop coating allows precise layering but may result in ring-like deposits, depending on variables [110,111,112]. Field-driven assembly uses magnetic and electric fields but may have miniaturization limitations [101,139,140]. Optical tweezers provide precise control but require sophisticated equipment [108,156,157,158,159,160,161].

While static and dynamic methods have shown promise in laboratory settings, scaling these methods to industrial levels poses a significant challenge. This is due to the intricate control required at the atomic or molecular level, and the complexity increases exponentially with the scale of the operation [10].

The financial implications of these methods are also a major concern. The high cost of the processes, particularly those requiring precise control and expensive equipment, can be a limiting factor for their widespread adoption. Moreover, the cost effectiveness of these methods needs to be evaluated in comparison with traditional manufacturing processes. Another challenge lies in the compatibility of these methods with various biological materials. While some materials may respond well to these methods, others may not be suitable due to their inherent properties [17]. Furthermore, the interaction of assembled particles with biological systems needs to be thoroughly investigated to ensure safety and efficacy. Despite these challenges, the field of particle assembly holds immense potential for the future. Continued research and development in this area are expected to address these issues and pave the way for new breakthroughs in biomaterial sciences [101].

Emerging technologies such as 3D printing at the nanoscale are showing promise in addressing these challenges. For instance, recent advances in the 3D printing of nanocellulose have facilitated a deeper understanding of their desirable attributes, such as high surface area, biocompatibility, and ease of functionalization. This additive manufacturing technique of new nanocellulosic materials has been developed to further reduce the carbon footprints and wastage of valuable resources. Moreover, a new 3D nanoprinting technique has been developed that works by depositing metal ions onto a negatively charged substrate to produce tiny metal objects. This technique has potential applications in microelectronics, sensor technology, and battery technology. These emerging technologies are expected to address the challenges in scalability, cost, and material compatibility, and pave the way for new breakthroughs in the field of particle assembly [26].

Emerging technologies such as machine learning are showing promise in addressing these challenges. For instance, machine learning algorithms have been used for the design optimization of electromagnetic devices. These algorithms can help by strongly reducing the overall computational times, making the use of complex simulation systems within the optimization cycle possible. Moreover, convolutional neural networks (CNNs), a type of machine learning algorithm, have been applied to material design problems. With the capacity to capture features at different hierarchical levels, CNNs are well suited to describe the properties of materials, especially biomaterials. These emerging technologies are expected to address the challenges in scalability, cost, and material compatibility, and pave the way for new breakthroughs in the field of particle assembly [246].

Looking toward the future, the potential applications of these methods in personalized medicine, regenerative medicine, and diagnostics are immense. For instance, the aim of personalized medicine is to detach from a “one-size fits all approach” and improve patient health by individualization to achieve the best outcomes in disease prevention, diagnosis, and treatment. Technological advances in sequencing, improved knowledge of omics, integration with bioinformatics, and new developments in particle assembly methods are expected to play a crucial role in this regard [26].

Regenerative medicine is a rapidly evolving field, with new developments in cellular therapeutics, extracellular vesicles (EVs), and tissue engineering strategies constantly emerging. These advancements are tailored to individual patients, offering a personalized approach to treatment. Numerous pre-clinical and clinical trials have showcased the immense potential of cellular therapies, including stem cells, immune cells, and EVs. These therapies can modulate inflammatory immune responses and stimulate neoangiogenic regeneration in various contexts, such as diseased organs, damaged grafts, and inflammatory diseases, including COVID-19 [9].

For diagnostics, the creation of nucleic acid-based receptors and aptamers, capable of detecting non-DNA/RNA molecules, opens attractive opportunities for tissue engineering. As these aptamers are refined and enhanced to bind to molecules, like metal ions and microenvironmental components, their detection capabilities are simultaneously improved through fluorescence-based or electrochemical modalities. Despite the challenges that lie ahead, the future of particle assembly within the framework of nanoarchitectonics for biomaterial sciences is promising. It holds potential for applications in personalized medicine, regenerative medicine, and diagnostics. The field continues to push the boundaries of what is possible, driving us toward a future where medicine is not just about treating symptoms but about healing from within [247].

## 8. Conclusions

This article offers an in-depth and enlightening examination of static and dynamic particle assembly methods within the realm of nanoarchitectonics for biomaterial sciences. It delves into the principles of these techniques, emphasizing their crucial role in the creation of nanomaterials of various sizes. The versatility of these methods is underscored, showcasing their wide-ranging applications in fields such as energy storage, supercapacitors, sensors, electromagnetic interference shielding, water purification, and bio-related uses. The potential of the nanoarchitectonics approach to fulfill societal needs in areas like energy, environment, and medicine is also accentuated. This thorough review acts as a crucial resource for both researchers and practitioners in the field. It clarifies how the principles of static and dynamic particle assembly methods can be utilized to create nanomaterials of different dimensions, thus expanding their application scope.

The significance of nanoarchitectonics is further emphasized by its potential to cater to societal needs in various sectors such as energy, environment, and medicine. By facilitating the production of functional material systems, nanoarchitectonics lays the groundwork for progress in biomaterial sciences, establishing itself as an essential instrument in this field. This article stands as a testament to the transformative power of nanoarchitectonics, spurring further investigation and innovation in the field.

Looking forward, it is suggested that research persistently explores and refines these methods, concentrating on the development of more effective and adaptable techniques for nanomaterial synthesis. The potential ramifications of these advancements in healthcare and technology are immense. In healthcare, enhanced nanoarchitectonics could result in more efficient drug delivery systems, improved diagnostic tools, and innovative treatments for a variety of diseases. In technology, the creation of more advanced nanomaterials could bring about a revolution in sectors such as energy storage, environmental science, and electronics.

However, as we press on, it is vital to consider the ethical and safety implications of these technologies. Future research should also strive to address these issues, ensuring that the benefits of nanoarchitectonics can be harnessed without compromising safety or ethical norms. This will guarantee that the field of nanoarchitectonics continues to make a positive contribution to society and human well being.

## Figures and Tables

**Table 1:** Summary of the advantages and disadvantages of the static and dynamic assembly methods.

Category	Assembly Method	Advantages	Disadvantages	
Static and dynamic	Self-Assembly	Uses weak interactions (e.g., van der Waals forces, hydrogen bonding) for the organization of nanoparticles	Limited in performing specific advanced functions due to conventional molecules’ limitations	[10,18,19,20,21]
Static	Layer-by-Layer Assembly	Exceptional control and versatility, applicable in various fields, allows the formation of multilayered structures	The complex physical mechanisms governing adsorbed quantities require a specifically charged substrate	[51,52,53,58,59,60,61,62]
	Template-Assisted Assembly	Precise assembly of nanoparticles, high degree of structural regularity, versatile applications	Requires specialized substrates or templates for organization	[2,10,16,29,70,72,73,74,75]
	Polymer Brushes	Can engage nanoparticles without aggregation, offers adjustability in properties	Weak interaction between polymer brushes and nanoparticles may lead to aggregation	[83,84,85,86]
Dynamic	Drop Coating	Offer precisely controlled, thin layers on a wide range of substrates. Versatile applications like thin films, protective coatings, and the deposition of materials onto surfaces. Homogeneous deposition of particles and control over particle distribution	The evaporating liquid can leave behind a ring-like deposit of particles. Dependency on variables, such as temperature and ethanol concentration, and substrate roughness	[110,111,112]
	Microfluidic Devices	Suitable for handling minute quantities of fluids, applicable in drug delivery systems, sensors, and pumps	Handling and control of fluids in microchannels can be challenging	[110,115]
	Capillary Assembly	Employs capillary forces for precise positioning of components, applicable in microelectronics and optics	Specialized technique requiring precise control of capillary forces	[118,119]
	Dip Coating	Simple, cost effective, and suitable for creating thin-film coatings	Control of coating thickness can be challenging; a potential slowdown in production due to drying and curing times	[120,121,122]
	Spin Coating	Widely used in semiconductor manufacturing, can produce films with thicknesses of less than 10 nanometers	Limitations due to specific assumptions in the dynamic method; factors like solvent diffusion and substrate surface roughness	[123,124,126,127,128,129,130,131]
	Langmuir–Blodgett	Precise method for assembling monolayers into nanostructured films, instrumental in surface science and nanotechnology	Not included in the primary focus of the study; relevance is limited to specific applications	[132,133,134]
	Freeze Casting	Forms materials with high porosity and complex geometries, useful in fabricating nanocomposites	Not included in the primary focus of the study; precision required in packing and pore directionality	[135,136,137,138]
	Magnetic Field	High precision in the manipulation of biomolecules and polymers, plays a pivotal role in biophysics and nanotechnology	Requires understanding of electromagnetism; limited approach with single-pole tweezers	[26,101,178,179,180,181,182,183,184,185,186,187,188,189,190]
	Acoustic Field	Rapid parallel fabrication of objects from a solution, mechanical stability, and self-sustaining properties of assembled structures	Early-stage technology with potential long-term effects on cells; complex dynamics surrounding cell proliferation and differentiation	[177,197,198,199,200,201,202,203,205]
